# Integrated proteome and phosphoproteome analysis of gastric adenocarcinoma reveals molecular signatures capable of stratifying patient outcome

**DOI:** 10.1002/1878-0261.13361

**Published:** 2022-12-29

**Authors:** Xue Lu, Yunyun Fu, Lei Gu, Yunpeng Zhang, Antony Y. Liao, Tingting Wang, Bin Jia, Donglei Zhou, Lujian Liao

**Affiliations:** ^1^ Shanghai Key Laboratory of Regulatory Biology, School of Life Sciences East China Normal University Shanghai China; ^2^ Department of General Surgery, Shanghai Tenth People's Hospital, School of Medicine Tongji University Shanghai China; ^3^ University of California San Diego La Jolla CA USA; ^4^ Durbrain Medical Laboratory Hangzhou China; ^5^ Department of Oncology The First Affiliated Hospital of Zhengzhou University China; ^6^ Department of Gastric Surgery Fudan University Shanghai Cancer Center China; ^7^ Department of Oncology, Shanghai Medical College Fudan University Shanghai China

**Keywords:** biomarker, gastric adenocarcinoma, lymph node metastasis, SPON1, TNXB

## Abstract

Metastasis is one of the main causes of low survival rate of gastric cancer patients. Exploring key proteins in the progression of gastric adenocarcinoma (GAC) may provide new candidates for prognostic biomarker development and therapeutic intervention. We applied quantitative mass spectrometry to compare the proteome and phosphoproteome of primary tumor tissues between GAC patients with and without lymph node metastasis (LNM). We then performed an integrated analysis of the proteomic and transcriptomic data to reveal the molecular features. We quantified a total of 5536 proteins, and we found 218 upregulated and 49 downregulated proteins in tumor samples from patients with LNM compared to those without LNM. Clustering analysis identified a number of hub proteins that have been previously shown to play important roles in gastric cancer progression. We also found that two extracellular proteins, TNXB and SPON1, are overexpressed in patients with LNM, which correlates with poor survival of GAC patients. Overexpression of TNXB and SPON1 was validated by western blotting and immunohistochemistry. Furthermore, treating gastric cancer cells with anti‐TNXB antibody significantly reduced cell migration. Finally, quantitative phosphoproteomic analysis combined with activity‐based kinase capture revealed a number of activated kinases in primary tumor tissues from patients with LNM, among which GSK3 might be a new target that warrants further study. Our study provides a snapshot of the proteome and phosphoproteome of GAC tumor tissues that have metastatic potential, and identifies potential biomarkers for GAC progression.

## Graphical abstract

We conducted an integrated proteomic analysis of primary tumor tissues from gastric adenocarcinoma (GAC) patients. We discovered overexpression of TNXB and SPON1 in patients with lymph node metastasis, which correlates with poor survival of GAC patients. Inhibiting TNXB function using specific antibody significantly reduced gastric cancer cell migration. Phosphoproteomic analysis combined with activity‐based kinase capture revealed abnormal activation of GSK3.

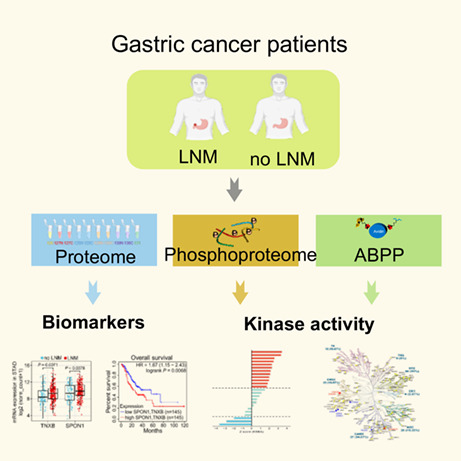

**Abbreviations**
ABPP: activity‐based protein profiling
ACN: acetonitrile
BLCA: bladder cancer
BRCA: breast cancer
bRP: basic reversed‐phase
CESC: cervical cancer
COAD: colon cancer
DAB: diaminobenzidine
DEGs: differentially expressed genes
DEPs: differentially expressed proteins
ECM: extracellular matrix
FDR: false discovery rate
FFPE: formalin‐fixed paraffin‐embedding
GAC: gastric adenocarcinoma
HCC: hepatocellular carcinoma
HRP: horse radish peroxidase
IHC: immunohistochemistry
IS: internal standard
lncRNA: long noncoding RNA
LNM: lymph node metastasis
MCC: maximal clique centrality
MCP: matricellular protein
m‐fuzz: fuzzy c‐means clustering
miRNA: microRNA
OS: overall survival
PCA: principal component analysis
PFI: progression‐free interval
PPD: Plasma Proteome Database
SPON1: spondin‐1
TEAB: triethylammonium bicarbonate
THCA: thyroid cancer
TMT: tandem mass tag
TNXB: tenascin‐X

## 1 Introduction

Gastric cancer is one of a leading cause of cancer‐related deaths worldwide [1], among which gastric adenocarcinoma (GAC) is the most common form [2]. Diagnosis of GAC is generally made at advanced stages when metastatic spreading occurs, which significantly reduces the efficacy of surgical treatment and contributes to poor patient survival [3, 4]. Accurate detection of GAC and prediction of cancer progression at early stages hold great promise to improve the clinical outcomes.

At initial diagnosis, lymph node metastasis (LNM) occurs in more than half of gastric cancer patients, leading to poor prognosis [5]. Therefore, lymph node status can be used as a powerful prognostic indicator for gastric cancer patients 5 years after radical surgery [6]. Early‐stage gastric cancer patients with four or more metastatic lymph nodes have a higher recurrence rate and lower survival rate [7]. LNM involves a series of complex biological processes including reduced adhesion to adjacent cells, degradation of extracellular matrix (ECM), and lymphatic channel infiltration [8]. ECM contains proteins, glycoproteins, and proteoglycans, and it plays a critical role in regulating cell–cell or cell–matrix interaction and influencing tumor progression [9]. Overexpression of matricellular proteins (MCPs), which are then secreted into the ECM, is an important feature during tissue remodeling [10]. Tumor cells secrete MCPs into the tumor microenvironment, thereby promoting tumor progression [11]. Therefore, capturing the molecular features in the premetastatic ECM is indispensable to understand the biological processes of tumor metastasis.

Protein kinases are important drug targets for many diseases. As of 2021, nearly 70 novel small molecule kinase inhibitors have been approved by FDA to combat various cancers. In 2021 alone, four kinase inhibitors were approved to treat cholangiocarcinoma, non–small‐lung cell carcinoma, and renal cell carcinoma [12]. While quantitative proteomics analyses depict the expression levels of kinases, quantitative phosphoproteomic studies reveal the activity of the kinases.

Recently, a number of reports analyzed the proteomic differences between tumor and para‐tumor tissues from gastric cancer patients, and explored the underlying molecular subtyping [13, 14, 15] as well as the correlation between identified protein markers and clinical outcomes of LNM [16, 17, 18, 19]. Nevertheless, many proteins may play important roles in promoting tumor progression and metastasis without showing differential expression between tumor and para‐tumor tissues. In addition, very little is known regarding the proteomic and phosphoproteomic features associated with gastric cancer progression and lymph node metastasis. Similarly, although phosphoproteomic analysis of gastric cancer has been conducted to compare tumor and para‐tumor tissues [20, 21], few studies have revealed kinase activity profiles in gastric tumors from patients with lymph node metastasis.

Here, we applied proteomic analysis to compare primary tumor tissues from GAC patients with or without LNM, and we captured unique molecular features of GAC patients with LNM. Analysis of the phosphoproteome provided a snapshot of abnormal phosphorylation signaling pathways and abnormal kinases activities. Furthermore, we found that TNXB and SPON1, two ECM proteins, are associated with LNM in GAC patients and play important roles in cancer cell migration. Thus, our study suggests a number of proteins and kinases that has the potential to serve as prognosis markers to predict patient outcome.

## 2 Materials and methods

### 2.1 Human samples

A total of 23 tumor and 22 para‐tumor frozen tissues from gastric adenocarcinoma (GAC) patients were collected from Shanghai Tenth People's Hospital. Except for one additional tumor tissue sample, each pair of tumor and para‐tumor tissues came from the same patient. All 45 tissue samples were analyzed using tandem mass tag (TMT)‐based quantitative proteomics and phosphoproteomics. *T* test (*P* value) and chi‐square test (*P* value*) are used to examine the clinical information, making sure that factors such as age and gender does not show statistical significance between tumor groups. The study conformed to the standards set by the Declaration of Helsinki and was approved by the Medical Ethics Committee of Shanghai Tenth People's Hospital (license number SHDSYY‐2020‐3645). The tissues used in the experiment were sampled from the surgically resected specimens from the patients, which would not cause additional pain and injury to the patients. Before the operation, each patient signed an informed consent form, informing them that the resected specimens will be used for pathological examination and related medical research.

### 2.2 TMT‐based proteomics analysis of tumor tissues

Frozen tissues were lysed in urea buffer (8 m urea, 50 mm Tris–HCl, pH = 8.0) containing 1% V/V protease and phosphatase inhibitor cocktail (Cat. #78440; Thermo Scientific, Waltham, MA, USA), using a tissue homogenizer (Cat. #JXFSTPRP‐24/32; Jingxin, Ningbo, China) with two porcelain beads per sample. Tissues were homogenized for 8 min (70 Hz, running time 30 s, stop time 10 s), followed by sonication for 10 min and centrifugation at 16 000 ***g*** for 10 min at 4 °C. The supernatant protein concentration was quantified by BCA (Cat. #23225; Thermo Fisher Scientific), then 150 μg proteins were reacted with 10 mm DTT at 55 °C for 30 min and alkylated with 15 mm iodoacetamide under dark for 20 min, followed by the overnight treatment of six times the volume of acetone for protein precipitation. Afterward, the buffer was exchanged to 100 mm Triethylammonium bicarbonate (TEAB, Cat. #140023; Sigma‐Aldrich, St. Louis, MO, USA), proteins were digested with trypsin at 37 °C for 12 h, and 1/10 of each sample was combined as an internal standard. The resulting peptides were labeled with 10‐plex tandem mass tag (TMT) reagent (Cat. #90110; Thermo Scientific) according to the manufacturer's instruction. After labeling for 1 h, 1% hydroxylamine was added to terminate the reaction. The labeled peptide samples in each batch of TMT labeling were then mixed, and 8% of each batch was used for quantitative proteomic analysis. Basic reversed‐phase (bRP) Kit (Cat. #84868; Thermo Fisher Scientific) was used for offline separation according to manufacturer's instructions. The LC‐MS/MS analysis methods were the same as previously described [22].

### 2.3 Enrichment of phosphorylated peptides

The remaining 92% of the TMT‐labeled peptide samples from each batch were separated by HPLC as previously described [20]. The sample was resuspended using basic reversed‐phase (bRP) buffer A (2% acetonitrile (ACN), 5 mm NH_4_COOH, pH = 10), eluted in 18–38% bRP buffer B (90% ACN, 5 mm NH_4_COOH, pH = 10) at a flow rate of 1 mL·min^−1^ for 60 min. In each minute, one fraction of the HPLC‐separated mixture was collected. After the samples were dried, the 60 fractions were resuspended with TiO_2_ sample buffer (80% ACN, 1% trifluoroacetic acid, 0.2 g phthalic acid·mL^−1^) and combined in a serpentine fashion into four fractions. TiO_2_ beads (Cat. #5020‐75000; GL Sciences, Tokyo, Japan) were activated with 2,5‐dihydroxybenzoic acid (DHB) buffer (80% ACN, 0.5% acetic acid, 0.02 g DHB·mL^−1^) for 30 min, and added into the sample to enrich phosphopeptides. The enrichment procedure was repeated once. The supernatant was then removed by centrifugation at 3500 ***g*** for 1 min, and the beads were sequentially washed with wash buffer 1 (5 mm KH_2_PO_4_, 30% ACN, 350 mm KCl), wash buffer 2 (40% ACN, 0.5% acetic acid, 0.05% TFA), and wash buffer 3 (80% ACN, 0.5% acetic acid), followed by sequential elution with 5% NH_3_·H_2_O and 10% NH_3_·H_2_O in 25% ACN. The eluent was dried and analyzed by LC‐MS/MS as described previously [22].

### 2.4 Activity‐based protein profiling of tumor tissues

KiNative kit (Cat. #88310; Thermo Fisher Scientific) was used for kinase enrichment. Primary tumor tissues from GAC patients with primary tumor and with LNM (*n* = 4) were lysed using buffer (25 mm Tris, pH = 7.6, 150 mm NaCl, 1% Tergitol NP‐40, 1% V/V protease and phosphatase inhibitor cocktail, 1 mm EDTA, 5% glycerol), followed by homogenization, sonication, and centrifugation as described above. Buffer‐exchange was performed using sepharose‐based gel filtration (Cat# 88310; Thermo Fisher Scientific), and the protein concentration was quantified by BCA. Then, 2 mg proteins were reacted with 10 mg of desthiobiotin‐ATP probe at a final concentration of 20 mm. All the experimental procedures were strictly following the manufacturer's instructions. The LC‐MS/MS analysis was following previously described procedures [22], except that each sample was analyzed in duplicate.

### 2.5 Proteomic data analysis

MS/MS spectra were searched using the maxquant (version 1.4.1.2, Max Planck Institute of Biochemistry, Planegg, Germany) software with a UniportKB human database. The precursor mass tolerance was set at 15 p.p.m., trypsin was set to the protease, and two missed cleavages were allowed. The false discovery rate (FDR) was set at 1% at both the peptide and protein level. For quantification, TMT 10‐plex (+229.1629 Da) on lysine and N terminus were added as static modifications. Serine, threonine, or tyrosine phosphorylation (+79.9663 Da) was set as variable modification, while oxidation of methionine (+15.9949 Da) and carbamidomethylation of cysteine (+57.0215 Da) were set as static modifications. For analysis of peptides modified by the KiNative probe, desthiobiotin on lysine residue (+196.121 Da) was set as variable modification.

The intensity of proteins or phosphorylation sites was first normalized by upper‐quantile, and then the samples were corrected by the internal standard within each TMT batch to remove batch effect. For differential expression analysis, proteins or phosphorylation sites with more than 50% missing values were removed. The expression of proteins or phosphorylation sites was tested for normality across all samples, then t‐test was applied for those with normal distribution, while Wilcoxon test was performed for those failed to pass normality test. In addition, differential expression analysis of phosphorylation sites was normalized to the expression of its proteins. r version 4.1.1 was used for most of statistical analyses.

For KiNative experiments, desthiobiotin‐modified lysine residues of kinases should cover lysine residues of kinase‐ATP binding site in Uniprot (https://www.uniprot.org/), including Lys1‐ATP binding site and Lys2‐active site [23]. After upper‐quantile normalization for all samples, the average intensity of desthiobiotin sites in two technical replicates was taken, and then the abnormal activity of kinases was analyzed by *t*‐test.

### 2.6 Transcriptome data analysis

limma package (version 3.48.3) [24] in r was performed for differential gene expression analysis comparing patients with primary tumor (T1‐4N0M0) and patients with LNM (T1‐4N1‐3M0). Data were downloaded from The Cancer Genome Atlas (TCGA) gastric adenocarcinoma datasets (Dataset ID: TCGA.STAD.sampleMap/HiSeqV2), TCGA breast cancer datasets (Dataset ID: TCGA.BRCA.sampleMap/HiSeqV2), TCGA colon cancer datasets (Dataset ID: TCGA.COAD.sampleMap/HiSeqV2), TCGA bladder cancer datasets (Dataset ID: TCGA.BLCA.sampleMap/HiSeqV2), TCGA cervical cancer datasets (Dataset ID: TCGA.CESC.sampleMap/HiSeqV2), TCGA thyroid cancer datasets (Dataset ID: TCGA.THCA.sampleMap/HiSeqV2). The age of the patients was between 40 and 90 years old.

### 2.7 GO and KEGG enrichment

Gene Ontology (GO) and Kyoto Encyclopedia of Genes and Genomes [25] enrichment analyses were performed using metascape (https://metascape.org/), David bioinformatics resource v6.8 (https://david.ncifcrf.gov/), and the package clusterprofiler [26] for r.

### 2.8 Protein–protein interaction and hub genes analysis

Analysis of protein–protein interaction network was performed using string (https://string‐db.org), with the minimum interaction score set to high confidence (0.7), and exported and constructed using cytoscape v3.9.0 (http://cytoscape.org).

Analysis of hub genes was performed using cytohubba (http://hub.iis.sinica.edu.tw/ciyohubba). Top 10 hub genes were calculated by the maximal clique centrality (MCC) method.

### 2.9 Mfuzz cluster analysis

mfuzz package [27] in r was applied to cluster proteins into six clusters based on samples from different stages of gastric cancer. Soft clustering was achieved by using the fuzzy c‐means algorithm. The membership value of a gene was between 0 and 1, indicating the degree of membership of the protein in a cluster.

### 2.10 Survival analysis

For Kaplan–Meier survival analysis, log‐rank test was applied to analyze differences in survival time [28]. The impact of gene expression on survival time was evaluated by the Cox proportional hazard model. Samples were stratified according to gene expression: samples with gene expression higher than 50% were considered as the high‐expression cohort, while samples with gene expression lower than 50% were considered as the low‐expression cohort. For survival analysis using TNXB and SPON1 as a panel, we used the intersection of the high‐expression cohort samples of the two genes as the new high‐expression cohort, and the intersection of the low‐expression cohort samples of the two genes as the new low‐expression cohort. The analysis was performed using r (4.1.1); TCGA stomach adenocarcinoma RNAseq dataset (Dataset ID: TCGA.STAD.sampleMap/HiSeqV2) and clinical information were downloaded from the UCSC genome browser (https://xenabrowser.net/).

### 2.11 Kinase activity prediction and visualization of Kinome tree

Kinase activity was predicted using KSEA [29], kinase‐substrate dataset was selected as “PhosphoSitePlus + NetworKIN,” while “NetworKIN score cutoff” was set as 2.5, “*P*‐value cutoff” was 0.05, and “substrate count cutoff” was 5. GPS5.0 (http://gps.biocuckoo.cn/) was used to verify downstream substrates of kinases, sequences of proteins were in FASTA format, and the threshold was set as “medium.” Kinome tree was conducted using kinmapbeta (http://www.kinhub.org/kinmap/).

### 2.12 Western blot

Frozen tissues were lysed by RIPA lysis buffer (150 mm NaCl, 0.5% SDC, 0.1% SDS, 50 mm Tris–HCl, pH = 7.6, 1.0% Triton X‐100, 1% V/V protease, and phosphatase inhibitor cocktail), followed by homogenized, sonication, and centrifugation as described above. The protein supernatant was added into 1 × Loading buffer and heated at 95 °C for 30 min. Subsequently, western blot was performed to protein expression as previously reported [22]. imagej and graphpad prism 8.0 (GraphPad Software Inc., San Diego, CA, USA) were applied to analyze the quantitative results with two‐tailed unpaired Student's *t*‐test. The antibodies used were anti‐TNXB (1 : 500, Cat. #13595‐1‐AP; Proteintech, Wuhan, China), anti‐SPON1 (1 : 200, Cat. #Bs‐7520R; Bioss, Woburn, MA, USA), anti‐ACTB/β‐actin (1 : 5000, Cat. #30101‐ES10; Yeasen, China), anti‐mouse IgG (H + L) (1 : 10 000, Cat. #33219ES60; Yeasen, Shanghai, China), and anti‐rabbit (1 : 10 000, Cat. #33119ES60; Yeasen).

### 2.13 Wound‐healing assay

Human gastric cancer cell lines BGC‐823 (RRID: CVCL_3360), SGC‐7901 (RRID: CVCL_VU58), HGC27 (RRID: CVCL_XB28), and MGC803 (RRID: CVCL_5334) were obtained from the cell bank of the School of Life Sciences, East China Normal University. Before the experiment, cell lines were authenticated by morphology evidence to avoid misrepresentation. All experiments were performed with mycoplasma‐free cells.

Cells were cultured in six‐well plates. A wound in the center of the well was introduced with a pipette tip when the cells were confluent. The culture medium was replaced with fresh RPMI 1640 medium without fetal bovine serum (FBS). The cells in the treatment group were treated with 0.1% TNXB antibody (Cat. #13595‐1‐AP; Proteintech), while the cells in control group were treated with sterilized ddH_2_O. Microscopic images were taken at the time point of 0 and 24 h.

### 2.14 Trans‐well migration assay

The 4 × 10^5^ cells in 200 μL RPMI 1640 medium without FBS were plated onto the upper layer of the trans‐well chamber (Cat. #3422; Corning, Tewksbury, MA, USA), while the lower layer contains 600 μL of the same culture medium with 20% of FBS. A 0.1% TNXB antibody (Cat. #13595‐1‐AP; Proteintech) was added to treatment group, while sterilized ddH_2_O was added to the control group. After 24 h, the cells were fixed with 4% paraformaldehyde for 20 min and then stained with crystal violet for 15 min. The unmigrated cells in the upper layer of the chamber were carefully wiped out with a cotton swab. Each chamber was imaged using an inverted microscope.

### 2.15 Cell viability assay

The 2 × 10^3^ cells in 100 μL RPMI 1640 medium with 10% FBS were added to each well of the 96‐well plate, and 10 μL of CCK‐8 reagent (Cat. #40203ES76; Yeasen) in 90 μL of RPMI 1640 medium was used to react with the cells. After 2 h, the cells were measured at a wavelength of 450 nm.

### 2.16 Immunohistochemistry

Immunohistochemistry was performed by IHC kit (DAB, rabbit, Cat#: ENS004; NeoBioscience, Shenzhen, China). Formalin‐fixed paraffin‐embedding tissues were dewaxed with xylene twice, xylene : ethanol = 1 : 1, 100%, 95%, 85%, 75%, and 50% ethanol for 3 min, respectively, following heat‐induced antigen retrieval in 100 °C EDTA for 20 min. Then, the tissue sections were immersed in 3% H_2_O_2_‐methanol solution for 10 min and washed with PBS. The solution was replaced with blocking buffer and incubated for 10 min, and then primary antibody was added and incubated for 1 h (anti‐TNXB, 1 : 50, Cat. #13595‐1‐AP; Proteintech; anti‐SPON1, 1 : 30, Cat. #TA351750; Origene, Rockville, MD, USA). After washing with PBS three times, horse radish peroxidase (HRP)‐conjugated anti‐rabbit secondary antibody (NeoBioscience) was added to the tissue and incubated for 10 min. After washing with PBS three times, HRP substrate solution was added and incubated for 10 min, followed by staining with DAB solution. Hematoxylin was used to stain nucleus for 5 min, then decolorized with hydrochloric acid, followed by dehydration by a series of 3‐min incubation using 50%, 75%, 85%, 95%, 100% ethanol, 50% xylene–ethanol solution, and finally xylene incubation twice, respectively. The tissue sections were then mounted and sealed on glass slides and examined under a microscope, with all images captured with identical camera exposure condition.

## 3 Results

### 3.1 Quantitative proteomic comparison of GAC tissues between patients with and without lymph node metastasis

We systematically explored the proteomic and phosphoproteomic profiles of GAC primary tumor and para‐tumor tissues comparing patients with and without lymph node metastasis (LNM). We collected 45 frozen tissue samples from 23 GAC patients, with pairs of primary tumor and para‐tumor tissues from each of the 22 patients, plus one tumor tissue from an additional patient. Based on the clinical information (Table 1; Table S1), 11 patients were diagnosed as primary tumor (designated “no LNM” thereafter) and 12 patients were diagnosed as advanced tumor with lymph node metastasis (designated “LNM” thereafter). The flow chart of the experiment is illustrated in Fig. S1.

**Table 1:** Clinical information

Characteristics		Tumor		Para‐tumor		Tumor, *P* value
		No LNM	LNM	No LNM	LNM	
Total cases		11	12	11	11	
Age (years)	Mean ± SD	66 ± 8.70	65 ± 11.35	66 ± 8.70	66 ± 8.20	0.7295
	Range	49–85	53–81	49–85	53–81	
Sex	Male	9 (81.8%)	10 (83.3%)	9 (81.8%)	9 (81.8%)	0.9336
	Female	2 (18.2%)	2 (16.7%)	2 (18.2%)	2 (18.2%)	
Stage	I	5 (45.5%)	0	5 (45.5%)	0	0.00006*
	II	6 (54.5%)	1 (8.3%)	6 (54.5%)	1 (9.09%)	
	III	0	11 (91.7%)	0	10 (90.9%)	
	IV	0	0	0	0	
T	1	4 (36.4%)	0	4 (36.4%)	0	0.1415*
	2	1 (9.09%)	1 (8.3%)	1 (9.09%)	1 (9.09%)	
	3	4 (36.4%)	7 (58.3%)	4 (36.4%)	6 (54.5%)	
	4	2 (18.2%)	4 (33.3%)	2 (18.2%)	4 (36.4%)	
	X	0	0	0	0	
N	0	11 (100%)	0	11 (100%)	0	0.00004*
	1	0	1 (8.3%)	0	1 (9.09%)	
	2	0	5 (41.7%)	0	5 (45.5%)	
	3	0	6 (50%)	0	5 (45.5%)	
	X	0	0	0	0	
M	0	11 (100%)	12 (100%)	11 (100%)	11 (100%)	—
	1	0	0	0	0	

* indicates Chi‐square test, otherwise: student's t‐test.

**Fig. 1:**
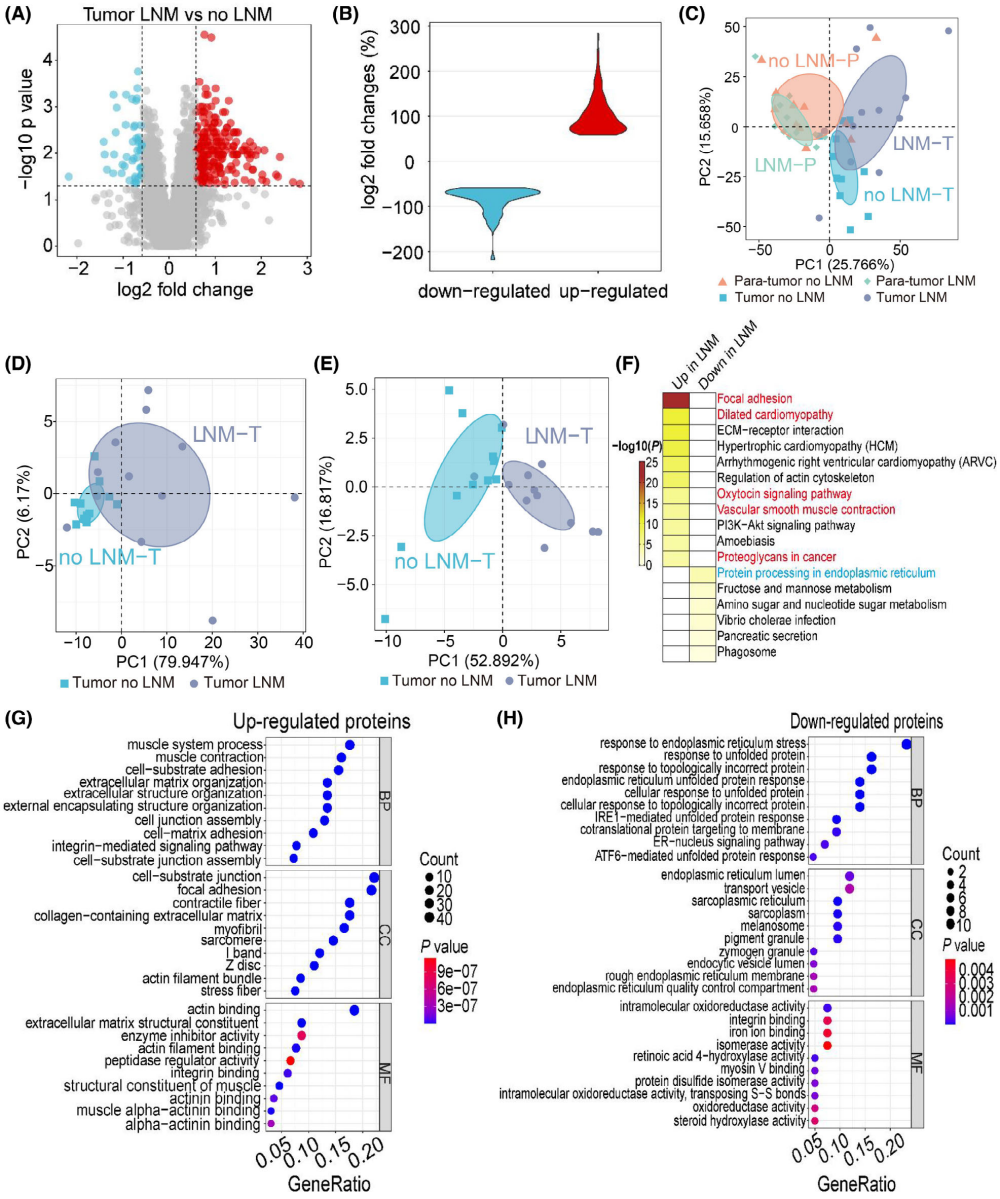
Quantitative proteomic comparison of tissues between gastric adenocarcinoma (GAC) patients with and without lymph node metastasis (LNM). (A) Volcano plot of differentially expressed proteins in primary tumor between GAC patients with LNM (*n* = 12) and without LNM (*n* = 11). Red dots represent significantly upregulated proteins with fold change ≥ 1.5, *P* value < 0.05. Blue dots represent significantly downregulated proteins with fold change ≤ 0.67, *P* value < 0.05. *T*‐test was applied for proteins with normal distribution, while Wilcoxon test was performed for those failed to pass normality test. (B) Violin plot of mean fold changes of down‐ and upregulated proteins. (C) Principal component analysis (PCA) of all samples, including tumor (*n* = 23) and para‐tumor tissues (*n* = 22), based on the intensity of all proteins. (D) PCA of tumor samples (*n* = 23) based on upregulated proteins. (E) PCA of tumor samples (*n* = 23) based on downregulated proteins. (F) Kyoto Encyclopedia of Genes and Genomes (KEGG) pathway analysis of differentially expressed proteins (DEP), showing pathways of upregulated proteins (left column) and downregulated proteins (right column). (G) Gene Ontology (GO) functional enrichment analysis of upregulated proteins. (H) GO functional enrichment analysis of downregulated proteins.

We applied high‐resolution quantitative mass spectrometry based on tandem mass tag (TMT) labeling for proteome and phosphoproteome measurements (Fig. S1, Table S1). In each of the five batches of labeling, we used the last channel as the internal standard (IS) to account for batch effects. The accuracy of the measurement is demonstrated by high correlation of the protein expression among the IS channels (Fig. S2A). The dynamic range of the signal intensity spans at least six orders of magnitude (Fig. S2B). In total, we quantified 5536 proteins from the 45 samples (Fig. S2C, Table S1). We normalized the data first by upper‐quantile intensity of each sample and then by the internal standard of each batch, resulting in a significant reduction of signal fluctuation among samples (Fig. S2D–F). Quantitative analysis revealed that there were 218 and 49 proteins significantly up‐ and downregulated in the LNM samples comparing to no LNM samples (Fig. 1A; Table S2). The overall fold change of differentially expressed proteins (DEPs) in tumor tissues was displayed in a violin diagram (Fig. 1B).

After correction for batch effects, principal component analysis (PCA) using the expression levels of all proteins separated the four types of samples relatively well (Fig. 1C), with the first component explaining nearly 25.8% of the variation, while the second component explaining 15.7%. That the first component separates tumor from para‐tumor indicated that the differences in protein expression in primary tumors between patients with and without LNM were less than that between tumor and para‐tumor tissues. Further PCA analyses of the proteome data were performed based on increased (Fig. 1D) and decreased (Fig. 1E) proteins, respectively, and the results showed that the DEPs distinguished tumor samples with better power. KEGG pathway analysis of the DEPs indicated that the increased proteins mainly contributed to focal adhesion, ECM–receptor interaction, and PI3K‐Akt signaling, while the decreased proteins involved in protein processing in endoplasmic reticulum, fructose, and mannose metabolism (Fig. 1F; Table S2). Consistent with gene ontology analysis, upregulated proteins mainly involved in cell–substrate adhesion and extracellular matrix organization (Fig. 1G; Table S2). In contrast, downregulated proteins mainly contributed to endoplasmic reticulum stress response and unfolded protein response (Fig. 1H).

### 3.2 Identification of biomarkers for GAC prognosis using c‐means clustering and hub protein analysis

**Fig. 2:**
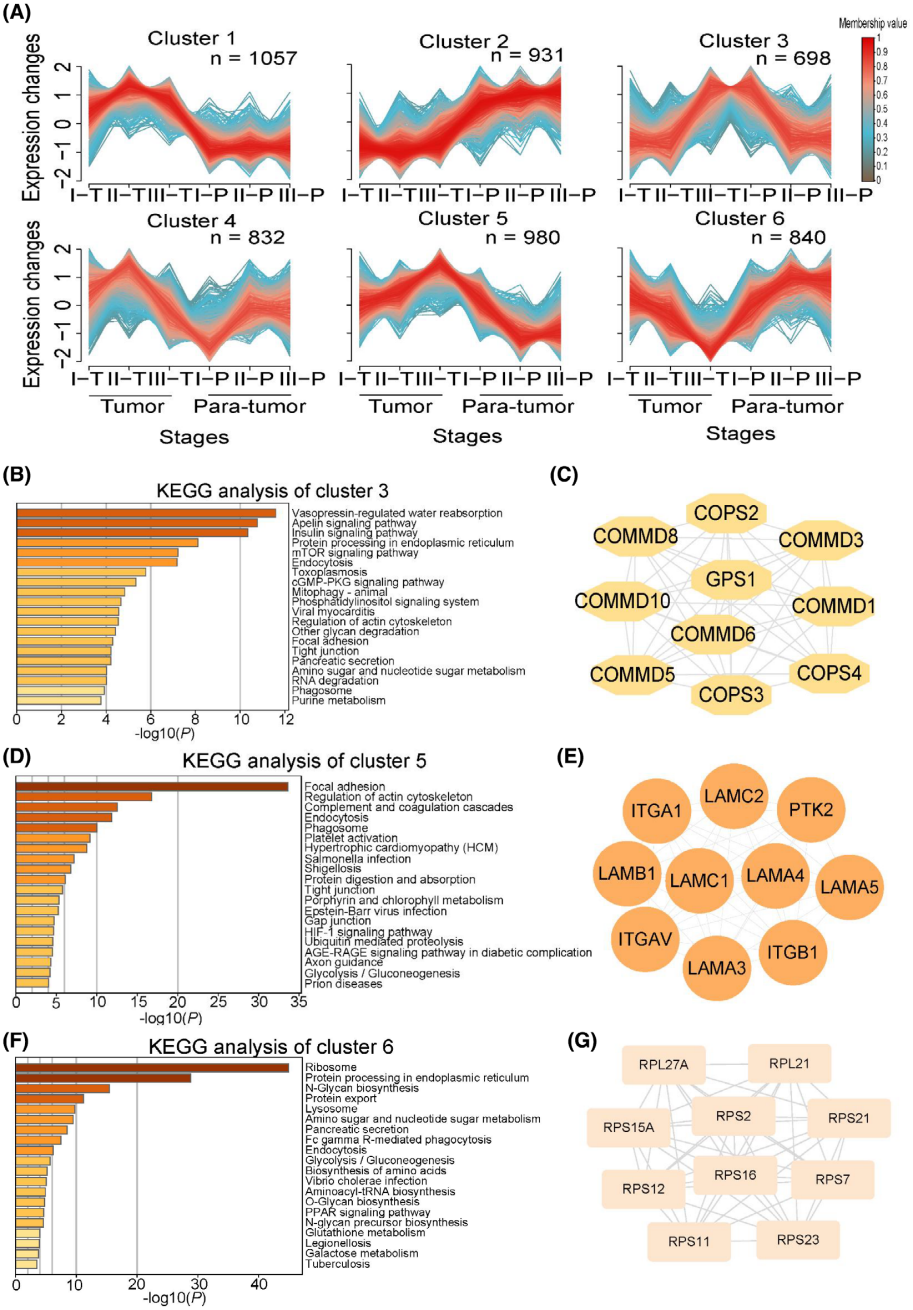
Clustering and network analysis of protein expression profiles related to GAC progression. (A) M‐fuzz identified six clusters of distinct protein expression patterns, comparing three tumor stages and two sample types (tumor and para‐tumor). (B) KEGG analysis of 698 proteins in cluster 3. (C) Top 10 hub proteins in cluster 3. (D) KEGG analysis of 980 proteins in cluster 5. (E) Top 10 hub proteins in cluster 5. (F) KEGG analysis of 840 proteins in cluster 6. (G) Top 10 hub proteins in cluster 6.

We then applied fuzzy c‐means clustering (m‐fuzz) to capture the dynamic protein expression among different stages of GAC, simultaneously considering both tumor and para‐tumor tissues. The analysis resulted in six distinct clusters of temporal expression patterns (Fig. 2A; Table S2). We focused on clusters that showed a trend of change among the three tumor stages. Clusters 3, 5, and 6 fit into this criterion. KEGG analysis of the 698 proteins in cluster 3 revealed that apelin signaling, insulin signaling, and mTOR signaling pathways are enriched (Fig. 2B), and the top 10 hub proteins in this cluster included GPS1, COPS2, and COMMD10 (Fig. 2C). On the other hand, focal adhesion and regulation of actin cytoskeleton are enriched among the 980 proteins in cluster 5 (Fig. 2D), the top 10 hub proteins in this cluster included ITGB1, ITGAV, PTK2 (FAK), and LAMA4 (Fig. 2E). For cluster 6, ribosome, protein processing in endoplasmic reticulum and *N*‐Glycan biosynthesis are enriched (Fig. 2F), the top 10 proteins included RPS2, RPS7, and RPS11 (Fig. 2G). Many of the hub proteins in these clusters have been shown to play a role in gastric cancer progression, including ITGB1, LAMA4, ITGAV, COMMD10, and LAMC1, and their expression at different GAC stages and Kaplan–Meier survival analysis related to each protein were shown in Fig. S3.

### 3.3 TNXB and SPON1 are biomarkers for lymph node metastasis in gastric adenocarcinoma

**Fig. 3:**
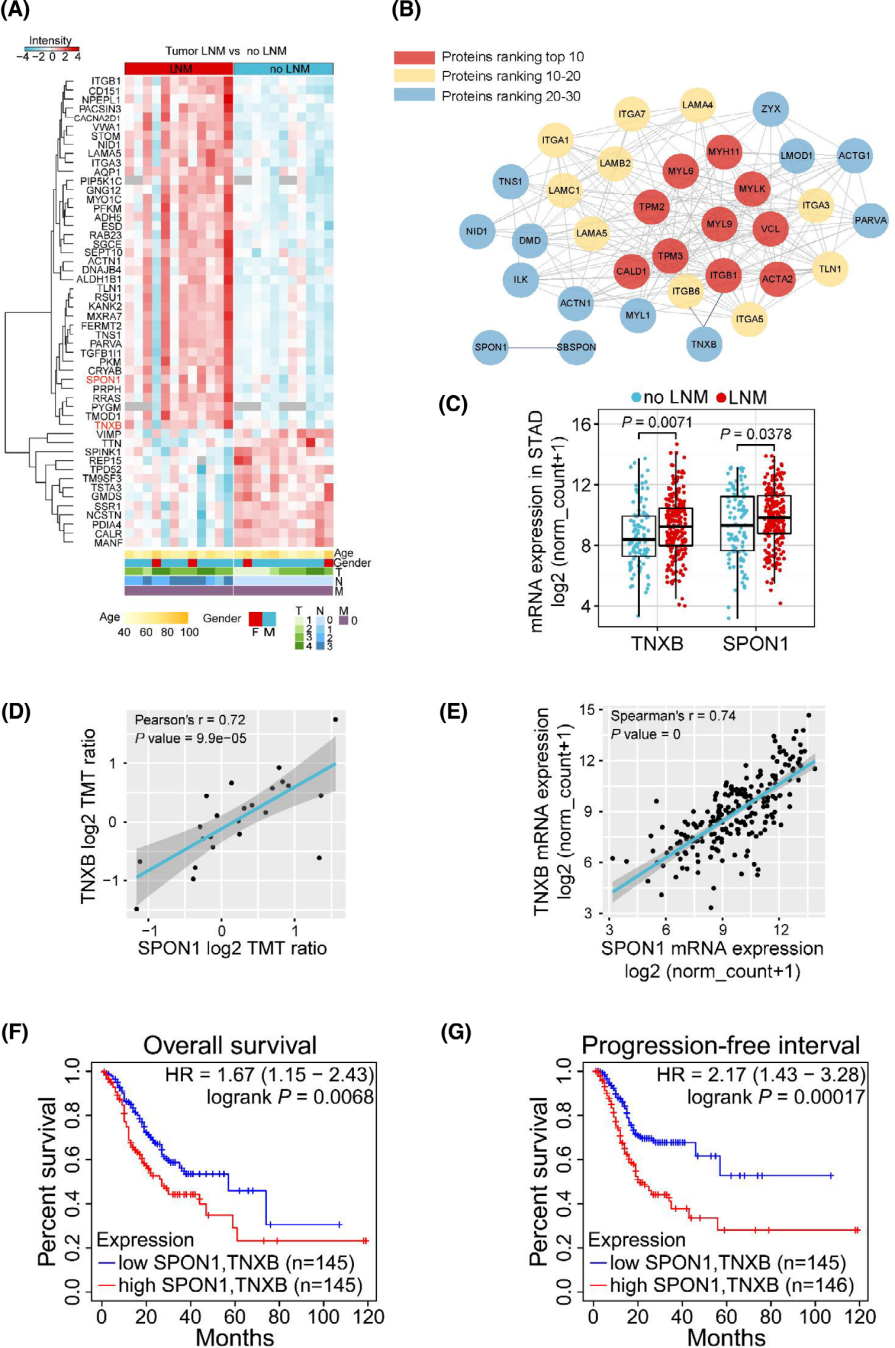
TNXB and SPON1 are potential biomarkers of lymph node metastasis in GAC. (A) Heatmap of top 50 most significantly changed proteins (smallest *P* value ranking) in primary tumors from GAC patients. (B) Protein–protein interaction network of top 30 upregulated proteins, including TNXB, SPON1, and their interaction proteins. (C) Expression of TNXB and SPON1 at mRNA level from The Cancer Genome Atlas (TCGA). Limma was used for differential gene expression analysis. (D) Correlation of protein expression between SPON1 and TNXB. Pearson's correlation was calculated. (E) Correlation of mRNA expression between SPON1 and TNXB from TCGA. Spearman's rank correlation was calculated. (F, G) Overall survival (F) and progression‐free interval (G) of GAC patients stratified by expression level of TNXB and SPON1 combined.

We used heatmap to display the top 50 most significantly changed proteins out of the 267 differentially expressed proteins (DEPs) in primary GAC tissues from patients with LNM (Fig. 3A). Comparing to the Plasma Proteome Database (PPD), we found that 216 proteins have been detected in plasma or serum previously, including 187 up‐ and 39 downregulated proteins. We plotted these differentially expressed plasma proteins in another heatmap (Fig. S4; Table S2), and they may serve as valuable resources for future biomarker studies. We then used the String database (https://string‐db.org/) to explore the protein–protein interaction of the 218 upregulated proteins with high confidence score of 0.7, and we used cytohubba to calculate the top 30 ranked proteins by means of maximal clique centrality (MCC). cytoscape was applied to construct the interaction network of the 30 proteins (Fig. 3B). A number of proteins have been reported to play important roles in gastric cancer metastasis, such as ITGB1, ITGAV, and LAMA4 [30, 31, 32, 33, 34, 35]. To seek further evidence, we explored the upregulated proteins at the transcription level, utilizing TCGA stomach adenocarcinoma datasets (Dataset ID: TCGA.STAD.sampleMap/HiSeqV2) to analyze the differentially expressed genes (DEGs) in primary tumor tissues between patients with and without LNM. Interestingly, Spondin‐1 (SPON1) and Tenascin‐X (TNXB), two proteins that have not been associated with gastric cancer before, showed significant upregulation (Fig. 3C), consistent with our proteomic findings. Correlation analysis showed that the expression of TNXB significantly correlated with that of SPON1 at both protein and mRNA levels (Fig. 3D,E). The correlation was further verified in TIMER2.0 and GEPIA databases (Fig. S5A,B).

We then performed survival analysis to explore the correlation between TNXB or SPON1 expression and the survival of GAC patients. High expression of SPON1 showed significant correlation with poor overall survival (OS) and progression‐free interval (PFI) (Fig. S5C,D). Similarly, high expression of TNXB showed significant correlation with poor OS and PFI (Fig. S5E,F). Furthermore, when combining SPON1 and TNXB for a multivariate survival analysis, the OS and PFI showed better distinction and more statistical significance, with a lower *P* value and higher hazard ratio (Fig. 3F,G).

**Fig. 4:**
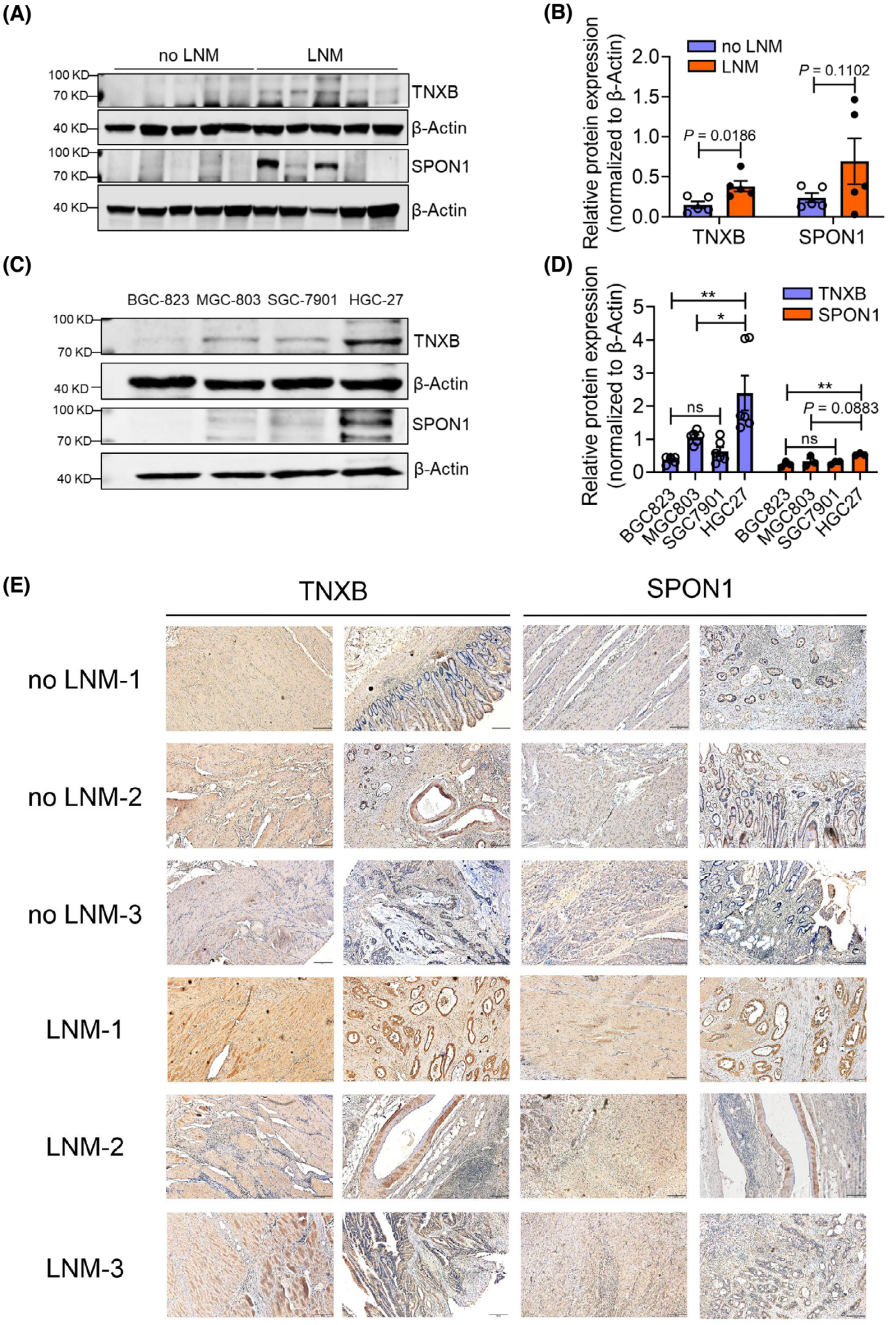
Validation of TNXB and SPON1 expression. (A) Western blot analysis of TNXB and SPON1 expression in tumor tissues. (B) Statistical analysis of the results shown in A (*n* = 5). (C) Western blot analysis of TNXB and SPON1 expression in gastric cancer cells. (D) Statistical analysis of the results shown in C (*n* = 3). *P < 0.05; ** *P* < 0.01; ns, no significance. (E) Immunohistochemistry validation of TNXB and SPON1 expression in FFPE samples. Tumor samples from three individual patients in each group were examined. Magnification: 100×, scale bar: 200 μm. For B and D: error bars indicate SEM, unpaired two‐tailed Student's *t*‐test.

To seek experimental evidence, we applied western blot analysis and confirmed higher expression of TNXB and SPON1 in GAC tumor tissues from patients with LNM (Fig. 4A,B). In addition, both proteins showed much higher expression in a highly aggressive, LNM GC cell line HGC‐27, than other two nonmetastatic GC cell lines BGC‐823 and MGC‐803, while another LNM GC cell line SGC‐7901 showed mild expression (Fig. 4C,D). Furthermore, we performed immunohistochemistry analysis (IHC) to examine the expression of TNXB and SPON1 in formalin‐fixed paraffin‐embedding (FFPE) primary tumor samples, and we found that there was overall stronger staining of TNXB and SPON1 in GAC tumor tissues from patients with LNM than from patients without LNM (Fig. 4E). Together, we confirmed that TNXB and SPON1 were upregulated in GAC tumor tissues from patients with LNM, thus substantiated our proteomic data.

We further explored the mRNA expression of TNXB and SPON1 in other types of cancer from TCGA, similarly comparing the differences in primary tumor samples from patients with or without LNM. The analysis showed that TNXB was upregulated in patients with bladder cancer (BLCA), cervical cancer (CESC), colon cancer (COAD), and breast cancer (BRCA) (Fig. S6A), while SPON1 was upregulated in patients with LNM in thyroid cancer (THCA) and bladder cancer (Fig. S6B). Thus, it is compelling to speculate that these proteins have the potential to serve as pan‐cancer markers for tumor spreading and metastasis.

### 3.4 TNXB promotes the migration of gastric cancer cells

**Fig. 5:**
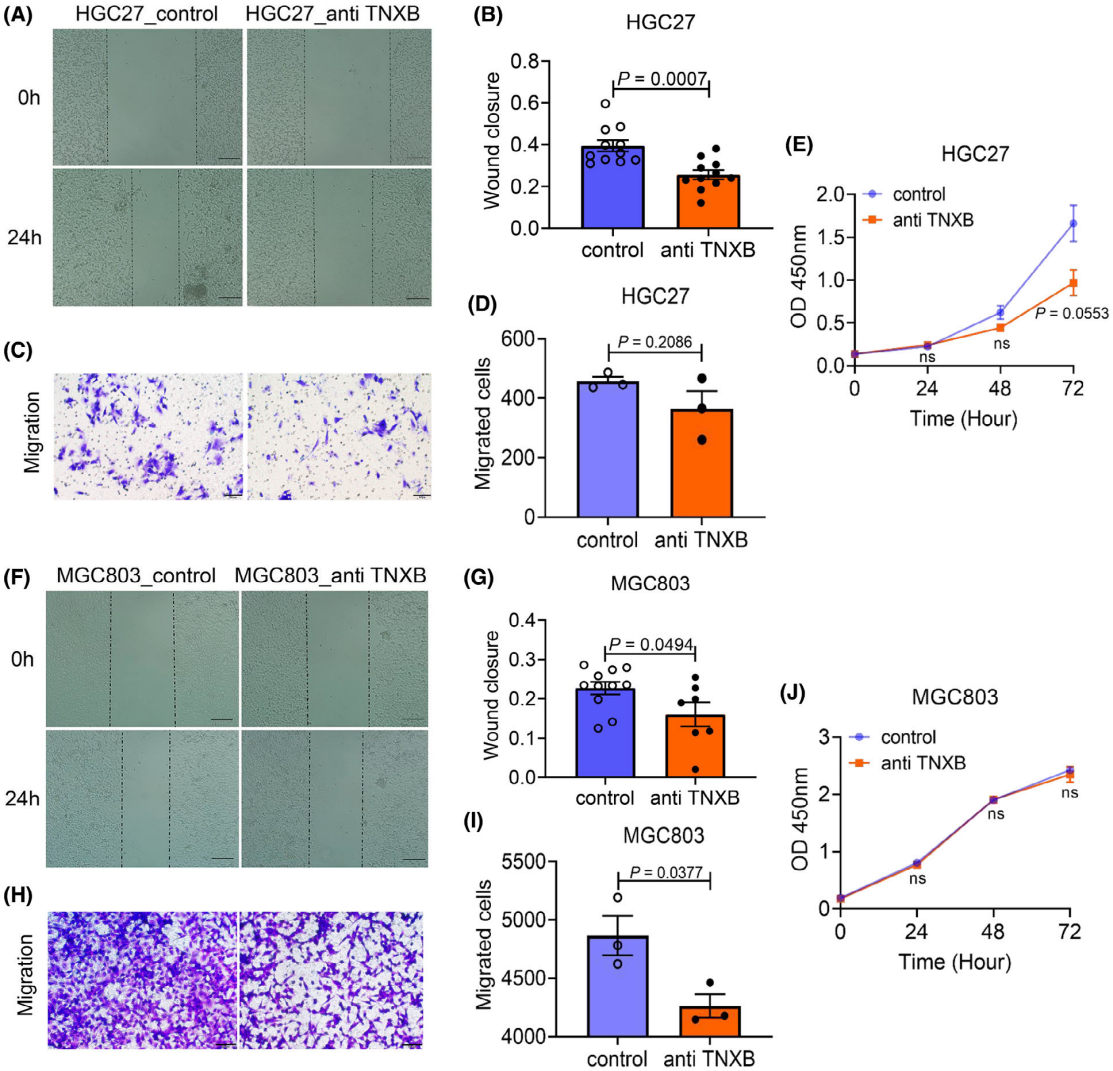
TNXB promotes migration of gastric cancer cells. (A) Wound‐healing assay of HGC27 cells examining the effect of anti‐TNXB antibody. Scale bar: 100 μm. (B) Statistical analysis of the results shown in A (*n* = 11). (C) Representative images of trans‐well assay examining the effect of anti‐TNXB antibody. Scale bar: 50 μm. (D) Statistical analysis of the results shown in C (*n* = 3). (E) Cell viability assay testing the effect of anti‐TNXB antibody on cell survival (*n* = 3). (F) Wound‐healing assay of MGC803 cells examining the effect of anti‐TNXB antibody. Scale bar: 100 μm. (G) Statistical analysis of the results shown in F (control, *n* = 11; anti‐TNXB, *n* = 7). (H) Representative images of trans‐well assay examining the effect of anti‐TNXB antibody. Scale bar: 50 μm. (I) Statistical analysis of the results shown in H (*n* = 3). Student's *t*‐test was used in all statistical analyses in this figure. (J) Cell viability assay testing the effect of anti‐TNXB antibody on cell survival (*n* = 4). For B, D, E, G, I, and J: error bars indicate SEM, unpaired two‐tailed Student's *t*‐test.

We selected two gastric cancer cell lines HGC27 and MGC803 in which TNXB was highly expressed to explore the function of TNXB in cell migration. In HGC‐27 cells, wound‐healing assay showed that TNXB antibody significantly reduced wound closure at 24 h (Fig. 5A,B). Although trans‐well assay failed to show statistical significance between cells with or without anti‐TNXB antibody treatment (*P* = 0.2086), there appeared to be a trend of reduced migration in cells after TNXB antibody treatment (Fig. 5C,D). TNXB antibody had minimal effect on cell proliferation at 24 or 48 h, although at 72 h, it significantly reduced proliferation (Fig. 5E). Similarly, in MGC‐803 cells, wound‐healing and trans‐well experiments also showed that TNXB antibody significantly reduced cell migration in both assays (Fig. 5F–I) without affecting cell proliferation (Fig. 5J). These results suggest that TNXB may promote tumor cell migration.

Protein–protein interaction analysis suggested that TNXB interacts with ITGB1 and ITGB6 (Fig. 3B). Increased expression of ITGB1 promotes invasion and migration of gastric cancer cells and is associated with poor prognosis and recurrence of GAC patients [36]. We went on to explore the correlation of expression between TNXB and ITGB1 and found that they show good correlation at both protein level (Fig. S7A) and mRNA level (Fig. S7B), a finding independently verified in two more databases GEPIA (Fig. S7C) and TIMER2.0 (Fig. S7D). These results suggest that TNXB promotes gastric adenocarcinoma progression potentially through interacting with ITGB1.

### 3.5 Relationship between TNXB, SPON1, and tumor immune microenvironment

Immune microenvironment plays an essential role in tumor occurrence and progression, with particularly important players are infiltrating immune cells including CD8+ and CD4+ T cells, macrophages, neutrophils, and dendritic cells. Therefore, we investigated the relationship between TNXB, SPON1, and immune cells in GAC using TIMER2.0. Expression of TNXB and SPON1 highly correlated with infiltrating macrophages and weakly correlated with neutrophils and myeloid dendritic cells (Fig. S8A). In addition, TNXB and SPON1 also displayed some correlation with CD4+ T cells, weak correlation with CD8+ T cells, and no correlation with B cells (Fig. S8B). These analyses suggested that TNXB and SPON1 may play important roles in tumor infiltrating immune cells, particularly macrophages, in gastric cancer.

In addition, we also analyzed the immune cell marker genes identified in our study. Nine differentially expressed proteins in primary GAC tissues from patients with LNM have been reported to be enriched in 24 types of immune cells [37]. Seven proteins showed significant upregulation, including THBS4, CMA1, CTSG, LDB3, KANK2, PDLIM4, and ALDH1B1. Two proteins including SULT1C2 and HPGD showed significant downregulation. TNXB showed significant positive correlation with THBS4, CMA1, LDB3, KANK2, PDLIM4, and ALDH1B1, while SPON1 showed significant positive correlation with all seven upregulated proteins. Thus, it appears that TNXB and SPON1 displayed certain degree of correlation with the tumor immune microenvironment (Fig. S8C).

### 3.6 Phosphoproteomic comparison of GAC tissues between patients with and without lymph node metastasis

**Fig. 6:**
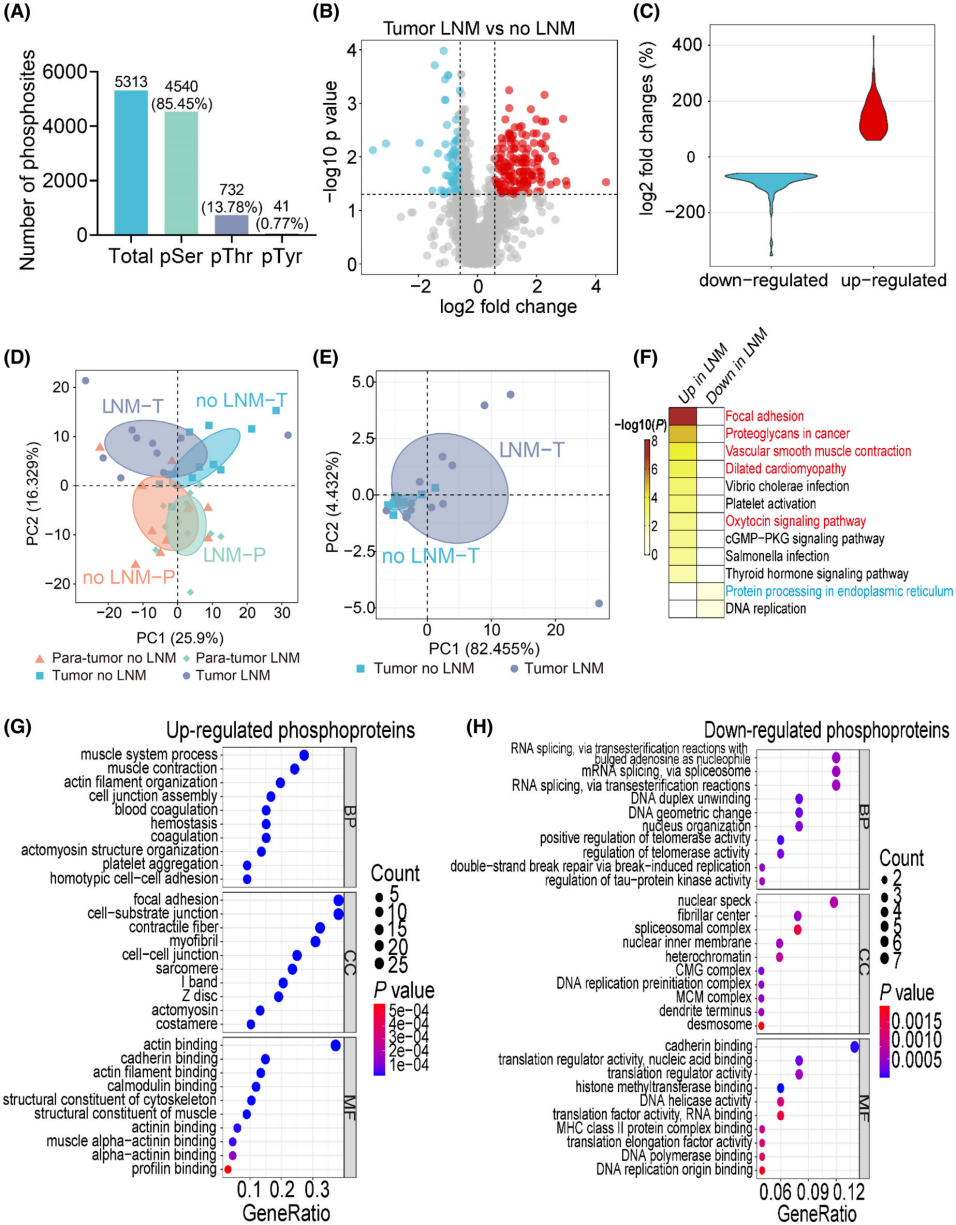
Phosphoproteomic comparison of tissues between GAC patients with and without lymph node metastasis. (A) Number and percentage of phosphorylation events on serine, threonine, and tyrosine residues (all samples, *n* = 45). (B) Volcano plot of differentially expressed phosphorylation sites in primary tissues between patients with or without LNM (no LNM: *n* = 11, LNM: *n* = 12). Red dots represent significantly upregulated phosphorylation sites with fold change ≥ 1.5, and blue dots represent significantly downregulated phosphorylation sites with fold change ≤ 0.67. *P* value < 0.05. (C) Violin plot of mean fold changes for down‐ and upregulated phosphorylation sites. (D) PCA of all samples based on all quantified phosphorylation sites (tumor: *n* = 23; para‐tumor: *n* = 22). (E) PCA of tumor samples based on upregulated phosphorylation sites (*n* = 23). (F) KEGG pathway analysis on changed phosphoproteins, showing KEGG pathways of upregulated (left column) and downregulated (right column) phosphoproteins. (G) GO functional enrichment analysis of upregulated phosphoproteins. (H) GO functional enrichment analysis of downregulated phosphoproteins.

Phosphoproteomic analysis of primary tumor and para‐tumor tissues identified 5313 phosphorylation sites, including 4520 serine (85.45%), 732 threonine (13.78%), and 41 tyrosine (0.77%) sites (Fig. 6A). Comparing to patients with only primary tumor, patients with LNM showed 118 upregulated and 78 downregulated phosphorylation sites (Fig. 6B; Table S3). The fold changes of differentially expressed phosphorylation sites were revealed by a violin diagram (Fig. 6C). PCA analysis revealed reasonable separation of tumor samples but not the para‐tumor tissue samples (Fig. 6D). Using upregulated phosphorylation sites, PCA showed better separation at the first component (Fig. 6E). KEGG pathway enrichment analysis for the differentially phosphorylated proteins revealed that they were mainly enriched in focal adhesion and proteoglycans in cancer (up), and protein processing in endoplasmic reticulum (down) (Fig. 6F, Table S3). GO functional enrichment analysis revealed that proteins with upregulated phosphorylation sites mainly involved in focal adhesion and acting binding, while proteins with downregulated phosphorylation sites mainly involved in RNA splicing (Fig. 6G,H; Table S3).

**Fig. 7:**
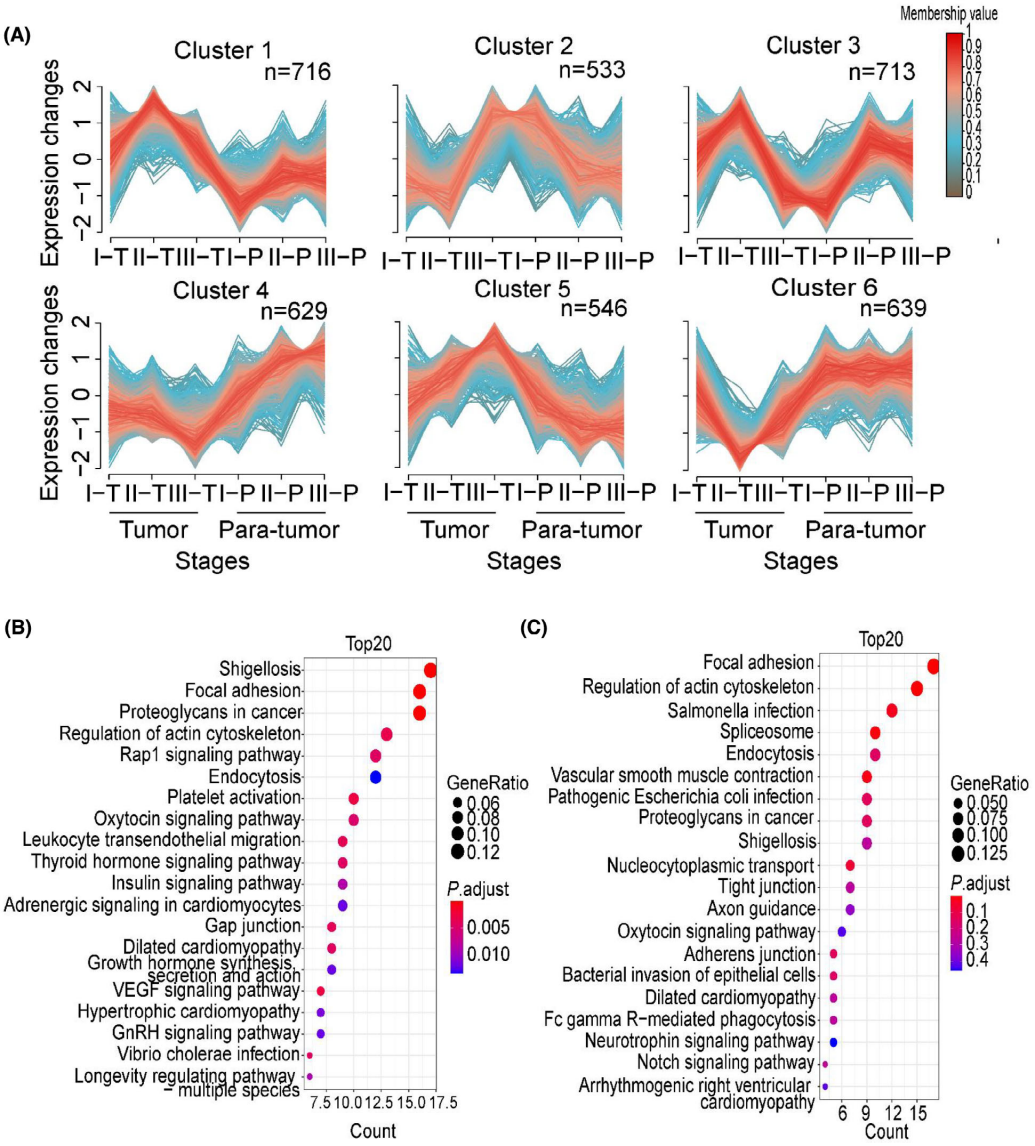
Changed phosphoproteins display distinct pattern during GAC progression. (A) M‐fuzz analysis of the quantitative phosphorylation results reveals six clusters, comparing three tumor stages and two sample types (tumor, *n* = 23, and para‐tumor, *n* = 22). (B) KEGG pathway analysis of phosphoproteins in cluster 2. (C) KEGG pathway analysis of phosphoproteins in cluster 5.

M‐fuzz analysis revealed a distinct expression pattern of six clusters (Fig. 7A; Table S3). Among these clusters, 533 phosphorylation sites corresponding to 313 phosphoproteins in cluster 2 showed a trend similar to cluster 3 in the proteome. KEGG pathway analysis also showed that these proteins mainly involved in focal adhesion, proteoglycans in cancer, and Rap1 signaling pathway (Fig. 7B). In addition, 546 phosphorylation sites corresponding to 320 phosphoproteins in cluster 5 exhibited a trend of increase in stages I–III tumor samples and decrease in the para‐tumor samples, which is similar to cluster 5 in the proteome. KEGG pathway enrichment further showed that most proteins contributed to focal adhesion, regulation of actin cytoskeleton pathways (Fig. 7C).

### 3.7 Abnormal activity of kinases in GAC primary tumor from patients with lymph node metastasis

**Fig. 8:**
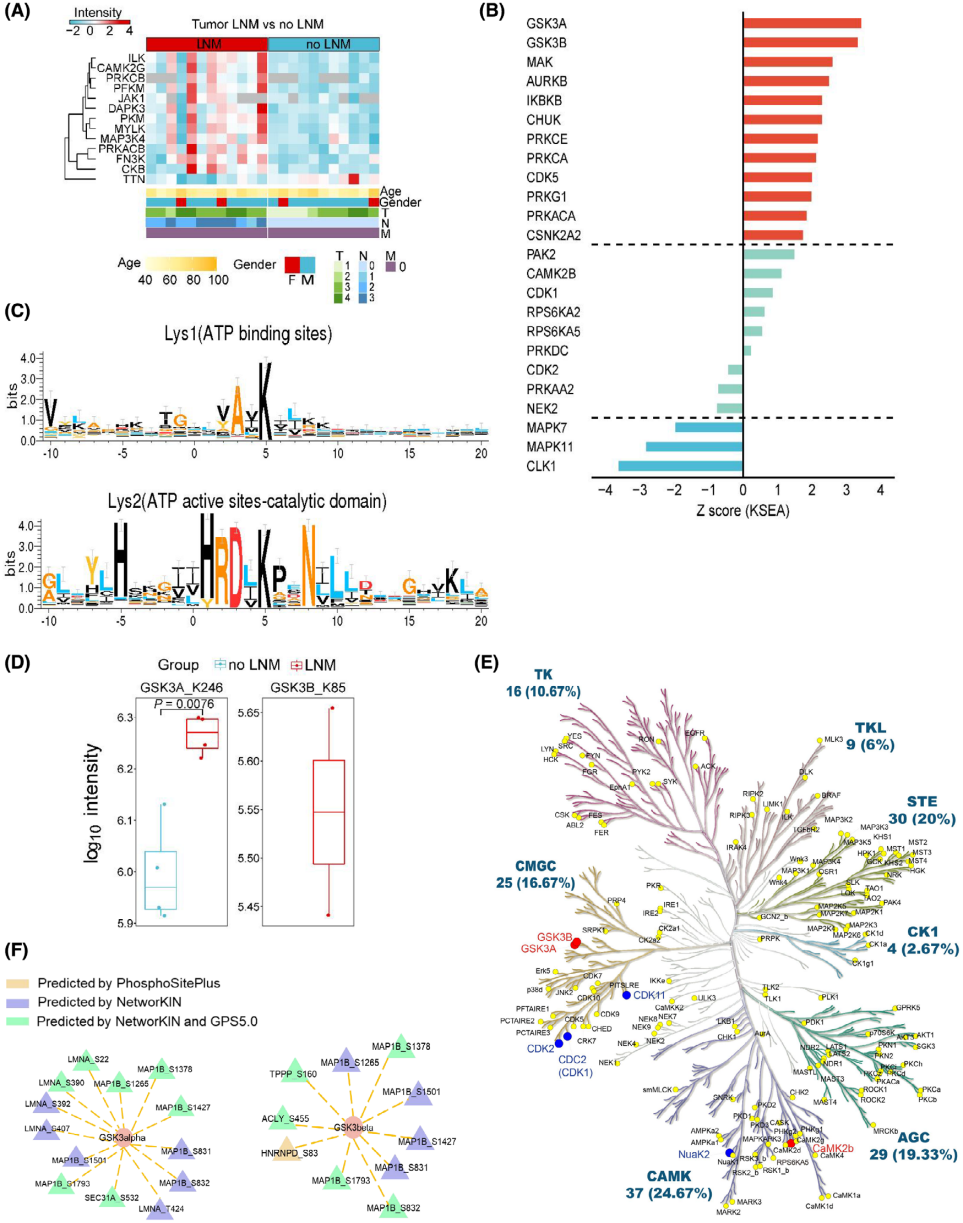
Changed kinase activity in GAC patients with lymph node metastasis. (A) Heat map representing differentially expressed kinases in primary tumor tissues from GAC patients with or without LNM. (B) KSEA predicted activity of kinases. Red and blue bars represent significantly upregulated and downregulated kinase activity (*P* value < 0.05), respectively, in patients with LNM comparing to without LNM. (C) Motif analysis of lysine‐binding sites of kinases captured by the ATP probe. (D) Box plot displaying the intensity of ATP probe‐modified peptides containing the lysine residue within the kinase activation pocket (*n* = 4), fold change ≥ 1.5 or ≤ 0.67, *P* < 0.05. (E) Kinome tree revealing kinases with changed activity in patients with LNM comparing to patients without LNM. Red dots represent kinases with significant increased activity, while blue dots represent kinases with significantly decreased activity in LNM. (F) A combined analysis using KESA and GPS5.0 reveals a kinase–substrate network, with the kinase locates at the center.

Protein kinases are important targets for small molecule inhibitor drug development. Thirteen kinases differentially expressed at the protein level in GAC tumor tissues with lymph node metastasis were plotted in a heatmap, including known drug targets JAK1, MAP3K4, and PRKCB, among others (Fig. 8A). We used KSEA [29] to predict the upstream activity of kinases that might contribute to the altered phosphorylation events, based on two databases PhosphoSitePlus and NetworKIN. Twelve kinases, including GSK3A, GSK3B, and MAK, were predicted to display significantly increased activity. On the other hand, three kinases were predicted to display significantly decreased activity, including CLK1, a previously reported therapeutic target in gastric cancer [38] (*P* < 0.05, Fig. 8B; Table S4).

We then conducted activity‐based protein profiling (ABPP) experiment to quantitatively capture kinases that are able to bind ATP, which are activated kinases [39]. Primary tumor tissues from four patients with LNM and four patients without LNM were used for the analysis. According to Uniport (https://www.uniprot.org/), ATP‐binding lysine residues inside the kinase activity pocket were annotated as either ATP‐binding site (Lys1) or active site (Lys2). Motif analysis of the ABPP probe‐modified lysine residues revealed that the −2 position of Lys1 was alanine, while the −2 position of Lys2 was aspartic acid (Fig. 8C; Table S4), consistent with a previous report [39]. When comparing kinase activity between patients with or without LNM, we found that the active site of GSK3A (K246) displayed a higher ATP‐binding ability in tumor samples from patient with LNM, consistent with the predicted results from KSEA. This suggests that GSK3A may be overactivated in tumors from patients with LNM (Fig. 8D). GSK3B (K85) was identified in two tumor samples from patients with LNM, suggesting its overactivation, which is in contrast to a prior study [40].

Kinome tree showed kinases with abnormal activity in GAC patients with LNM that were validated by ABPP (Fig. 8E). The kinase–substrate relationship was constructed using KSEA based on NetworKIN and PhosphoSitePlus (Table S4), which was further verified by GPS5.0 (http://gps.biocuckoo.cn/) using a medium threshold. The phosphorylation substrates that were validated in both GPS5.0 and NetworKIN were presented as green triangles, which were predicted to be the downstream phosphorylation substrates of the respective kinase indicated by orange ovals (Fig. 8F).

**Fig. 9:**
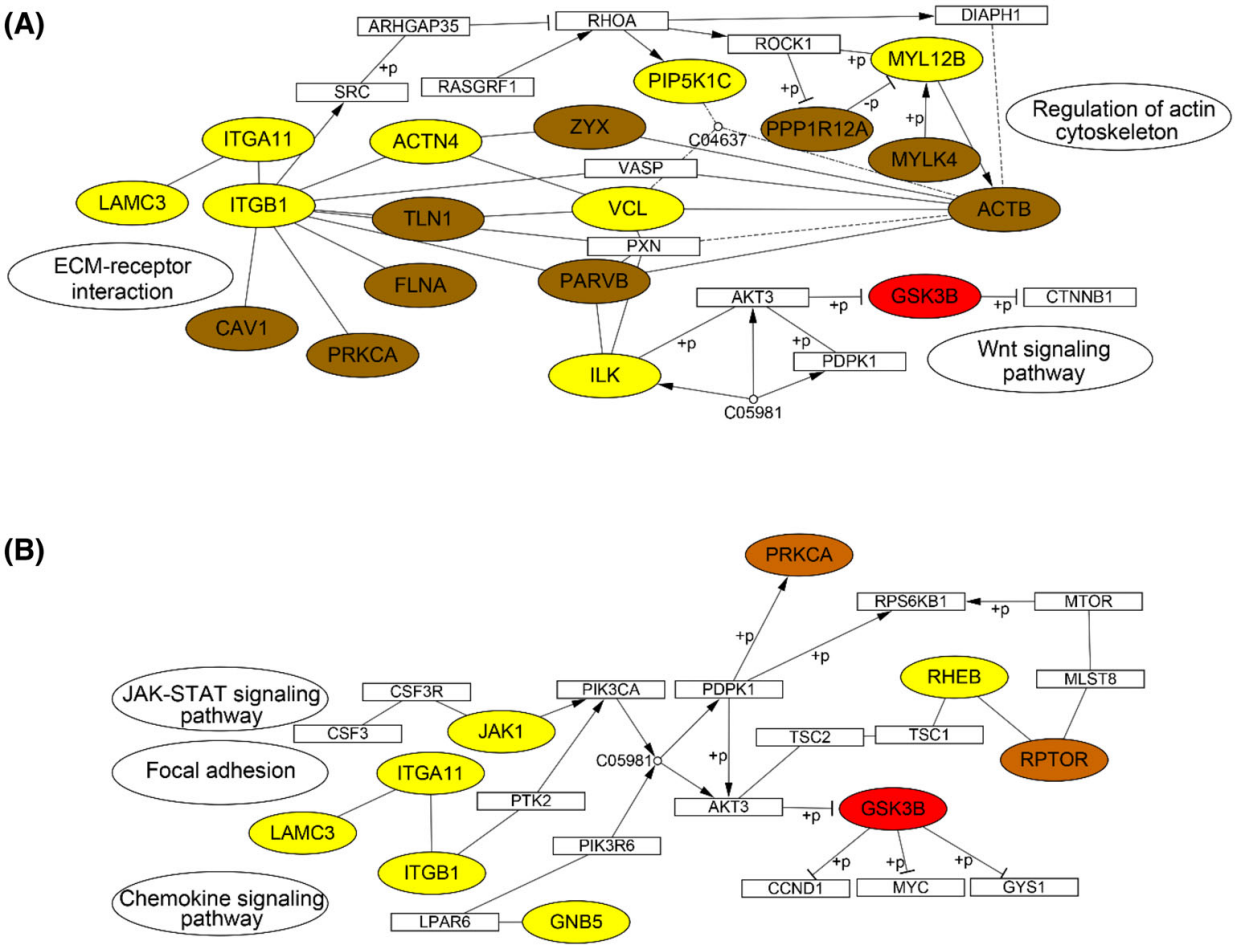
KEGG pathways of upregulated proteins (yellow), phosphoproteins (brown), and abnormal activity kinases (red). (A) Focal adhesion. (B) PI3K–AKT signaling pathway. “+p” denotes phosphorylation.

To reveal the molecular events underlying lymph node metastasis of GAC tumor, we utilized KEGG pathway analysis and mapped the upregulated proteins (yellow), phosphoproteins (orange), and kinases with abnormal activity (red) (Fig. 9). Consistent with alterations in the proteome, the reconstructed pathways revealed that focal adhesion and PI3K–AKT pathways were abnormally activated in GAC tumor tissues with LNM. The ECM proteins and integrins were also upregulated, involving integrin receptor binding to ECM proteins and leading to dimerization of focal adhesion kinase (FAK), which in turn led to autophosphorylation of FAK at Y397. The binding of SRC family kinases to the phosphorylation site of FAK forms the activated FAK–SRC complex [41]. FAK serves an important target for cancer progression, while FAK inhibitors have been under preclinical trial in combinatorial therapy [42]. Our study also found a number of activated signaling nodes in PI3K–AKT pathway. PI3K–AKT–mTOR pathway plays a significant role in progression of various cancers such as gastric cancer [43] and breast cancer [44]. Abnormal activation of PI3K–AKT–mTOR promotes cell survival by inhibiting apoptosis and also plays a role promoting metastasis [43].

## 4 Discussion

While a few previous reports investigated the correlation between abnormal proteins or kinases and lymph node metastasis in gastric adenocarcinoma, they primarily focused on comparing tumor tissues and adjacent tissues. It is difficult to identify biomarkers that may play important roles in GAC progression if they express at similar level between tumor and para‐tumor tissues. To the best of our knowledge, this study is the first comprehensive proteomic and phosphoproteomic comparison of GAC tumor tissues between patient with or without lymph node metastasis. Through analysis of upregulated proteins in tumor tissues from patients carrying LNM, we found that focal adhesion and ECM proteins are the most significantly enriched biological pathways, which faithfully captured the initial step of GAC tumor spreading and metastasis. Our further analysis identified overexpression of TNXB and SPON1 in GAC patients with LNM. Although these two proteins have been reported in other cancer types, but this study is the first to describe their abnormal expression in GAC tumors with LNM. Experimental validation and functional studies indicate that TNXB may be involved in gastric cancer cell migration and spreading. Finally, phosphoproteomic analysis combined with activity‐based kinase profiling identified abnormal activation of GSK3 in primary tumor tissues from GAC patients with LNM. Together, these results provide a valuable resource for a deeper understanding of molecular events dictating GAC tumor spreading and metastasis.

A number of proteins whose abnormal expression are consistent with previous reports. For example, we found that AQP1 was upregulated in tumors from patients with LNM, and the upregulation of AQP proteins has been reported to associate with lymph node metastasis, recurrence, and low overall survival rate in multiple cancers [45]. We also found overexpression of integrin family members including ITGB1 and ITGAV, which is associated with poor overall survival of gastric cancer patients. Both ITGB1 and ITGAV play important roles promoting the invasion and migration of gastric cancer cells *in vitro* and are associated with poor prognostic outcomes [30, 31]. Furthermore, overexpression of LAMA4 significantly impairs proliferation, invasion, and migration of ovarian, breast, and hepatocellular cancer cells [32, 33, 34]. We found that LAMA4 was increased during the progression of gastric adenocarcinoma, and its overexpression significantly correlated with poor overall survival of GAC patients, consistent with a previous report [35]. In addition, our data show for the first time that GPS1 is associated with the progression of gastric adenocarcinoma. GPS1 (also known as CSN1) is known to form a complex and play multiple roles in cellular and developmental processes including cell cycle regulation, DNA repair, and others. In hepatocellular carcinoma cells, GPS1 promotes cell proliferation and migration by upregulating the expression of cyclin A2 [46]. Thus, upregulated proteins in our proteomic data provide a rich resource for further understanding of GAC progression.

Our study found for the first time that TNXB and SPON1 are highly expressed in the primary tumor tissues of GAC patients with lymph node metastasis, and that overexpression of TNXB and SPON1 is associated with poor prognosis of GAC patients. When combining the two proteins, their power of predicting the prognosis of GAC patients was more significant. SPON1 is a cell adhesion molecule that promotes neurite extension and expresses at high levels in the floor plate [47], and has been shown to upregulate in other cancer types. SPON1 upregulation is closely related to metastasis and progression of osteosarcoma, promoting migration and invasion of osteosarcoma cells *in vivo* and *in vitro*, as well as activating FAK (PTK2) and SRC in osteosarcoma cells [48]. In addition, SPON1 is the direct target of miR‐506 via its binding to 3′‐UTR of SPON1 gene. Overexpression of miR‐506 suppressed hepatocellular carcinoma both *in vitro* and *in vivo* and reduced the accumulation of SPON1 both in hepatocellular carcinoma (HCC) cells and mouse tissues [49]. On the other hand, TNXB is an extracellular matrix (ECM) protein capable of direct interacting with a number of ECM molecules [50], playing different roles in different type of cancer. TNXB is overexpressed in malignant mesothelioma tumor tissues comparing to normal tissues, and it is also a new diagnostic marker of malignant mesothelioma [51, 52]. Tenascin X (TNX) plays a role in gastric function, while TNX‐deficient mice had significantly promoted vagal afferent response to gastric distention and accelerated gastric empties [53]. Taken together, our study suggested that TNXB and SPON1 may be potential biomarkers for GAC progression.

Phosphorylation of GSK3B at S9 affects the binding of GSK3B to substrates, and resulting in inhibition of the kinase. Previous studies have shown that S9 phosphorylation promotes the growth and migration of gastric cancer cells [54, 55], whereas constitutive activation of GSK3B correlated to better prognosis of gastric cancer patients [40]. In this study, we predicted kinase activity based on differential phosphorylation and experimentally verified the kinase activity using ABPP. We found that GSK3A and GSK3B are activated in GAC tumor tissues with lymph node metastasis. ABPP probes the binding of kinases with ATP at their conserved domains, which are generally the activation domain of the kinase. In the case of GSK3B, the activation residue locates at K85, and mutation of K85 reduced ATP binding of GSK3B, leading to reduced activity [56]. Our study found that GSK3B binds to ATP probe in two of four samples from tumor tissues of GAC with LNM, but not in the control group (Fig. 7D). This suggests an increased activity of GSK3B in tumor tissues from these patients. In addition, we found the upregulation of CD151 in the proteomic data, which is a known activator of GSK3B and positive regulator of β‐catenin expression, promoting osteosarcoma metastasis [57]. We postulate that GSK3B may potentially initiate this pathway to promote lymph node metastasis in gastric adenocarcinoma. On the other hand, there has been no report regarding the role of GSK3A in gastric cancer, although GSK3A was shown to promote the survival of lung cancer cells [58], and both GSK3A and GSK3B are abnormally activated in non‐type bladder cancer cells [59]. Thus, our study discovered that GSK3A and GSK3B are significantly activated in GAC primary tumor tissues from patients with lymph node metastasis, suggesting GSK3 may serve as a potential therapeutic target which warrants further validations.

The major limitation of this study is a relatively small sample size, which at present compromises the generalization of our findings in clinical settings. With dramatically improved sample size, one could perform independent and potentially multiple center validation experiments. When sample size reaches the scale of hundreds, one can also apply machine learning modeling, using TNXB, SPON1, and other differentially expressed proteins also found in plasma as biomarkers, to stratify patients between localized primary tumor and tumors with the tendency to metastasize to nearby lymph nodes. Another limitation of this study is that the exact mechanism of action of TNXB and SPON1 remains elusive. Based on the clues that both extracellular proteins interact with integrin family members, further studies could aim toward deciphering how these proteins interact with and promote integrin signaling. In perspective, further validation of potential protein biomarkers discovered in our study can identify GAC patients with higher probability of metastasis, guiding toward more aggressive treatment, and in turn dramatically improve the survival of GAC patients.

## 5 Conclusion

Our study identified molecular features for lymph node metastasis in GAC, including prognostic biomarkers TNXB and SPON1, potential kinase targets GSK3, as well as abnormal signaling pathways at the levels of protein expression and phosphorylation. In addition, we also identified prognostic markers and signaling pathways associated with progression of GAC by M‐Fuzz clustering. Overall, these results provide new targets for prognosis and potential treatment of gastric adenocarcinoma.

## Conflict of interest

The authors declare no conflict of interest.

## Author contributions

LL conceived the idea and directed the study. XL performed the majority of the experiments and analyzed the data. YF performed the majority of the bioinformatic and statistical analysis. XL and YF prepared the figures. YZ and TW contributed to some experimental data. AYL contributed to some data analysis and edited the manuscript. LG and DZ contributed to clinical samples. BJ contributed to clinical consultation. LL and XL wrote the manuscript.

### Peer review

The peer review history for this article is available at https://publons.com/publon/10.1002/1878‐0261.13361.

## Supporting information


**Fig. S1.** Schematic workflow of experiment design.

**Fig. S2.** Proteomic data assessment and preprocessing.

**Fig. S3.** Overall survival analysis of hub proteins.

**Fig. S4.** Heatmap of significantly DEPs in primary tumor from GAC patients in Plasma Proteome Database (PPD).

**Fig. S5.** (A) Correlation analysis of SPON1 and TNXB expression at transcriptional level in TIMER 2.0 dataset. (B) Correlation expression analysis of SPON1 and TNXB in GEPIA dataset. (C–D) Overall survival (C) and progression‐free interval (D) of GAC patients using Kaplan–Meier estimator stratified by SPON1 expression, using 50% as the expression cutoff. (E–F) Overall survival (E) and progression‐free interval (F) of GAC patients using Kaplan–Meier estimator stratified by TNXB expression, using 50% as the expression cutoff.

**Fig. S6.** (A–B) mRNA expression of TNXB (A) and SPON1 (B) in other types of cancer from TCGA database between patients with only primary tumor versus patients with LNM.

**Fig. S7.** (A–D) Correlation expression analysis of ITGB1 and TNXB at protein level (A), transcription level in TCGA dataset (B), GEPIA (C), and TIMER 2.0 (D).

**Fig. S8.** (A–B) Correlation of TNXB, SPON1 expression, and infiltrating immune cells in gastric adenocarcinoma based on ITMER 2.0 dataset, including macrophages, neutrophils, myeloid dendritic cells (A), and T cells CD4+, CD8+, B cells (B). (C) Correlation of TNXB and SPON1 with immune cell‐specific marker genes at the protein level. * *P* < 0.05, ** *P* < 0.01, *** *P* < 0.001, n.s. *P* > 0.05.

**Table S1.** Clinical information for proteomics, phosphoproteomics, and kinome.

**Table S2.** Differential expression analysis and KEGG enrichment of proteome, related to Fig. 1 and Fig. 2.

**Table S3.** Differential expression analysis and KEGG enrichment of phosphoproteome, related to Fig. 6 and Fig. 7.

**Table S4.** Prediction and validation of kinase activity based on phosphorylation and activity‐based protein profiling, related to Fig. 8.

## Data Availability

Data are available via ProteomeXchange with identifier PXD033622.
