# Supplementary material for: Integrated proteome and phosphoproteome analysis of gastric adenocarcinoma reveals molecular signatures capable of stratifying patient outcome
Source: Mol Oncol. 2022 Dec 29;17(2):261–83. doi: 10.1002/1878-0261.13361 (PMC9892830; doi:10.1002/1878-0261.13361)
Supplement: Supplementary file 1 — Fig. S1. Schematic workflow of experiment design. [file MOL2-17-261-s001.pdf]

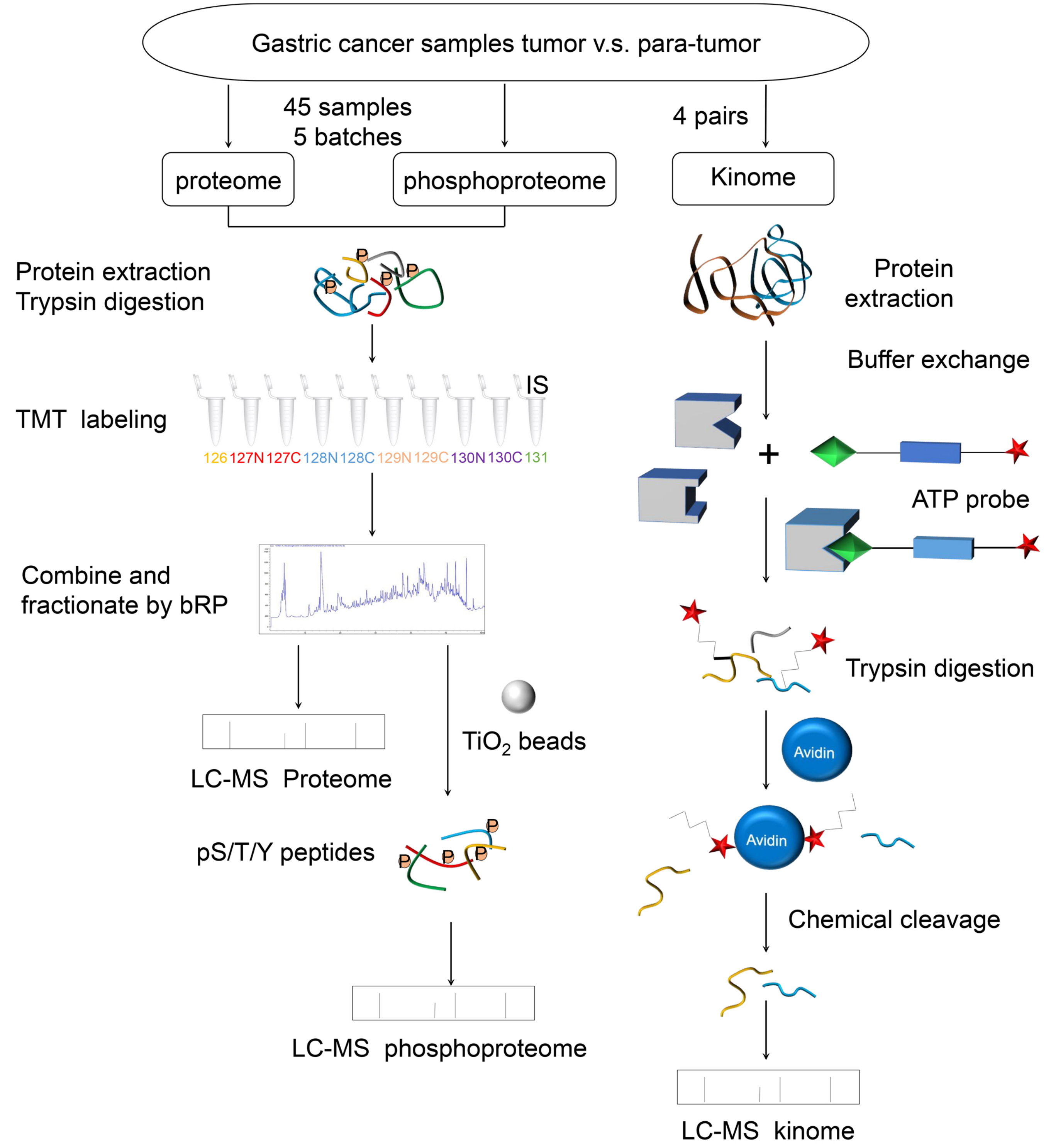
Gastric cancer samples tumor v.s. para-tumor
45 samples
5 batches
4 pairs
proteome
phosphoproteome
Kinome
Protein extraction
Trypsin digestion
Protein extraction
Buffer exchange
TMT labeling
IS
126 127N 127C 128N 128C 129N 129C 130N 130C 131
ATP probe
Combine and fractionate by bRP
Trypsin digestion
TiO2 beads
LC-MS Proteome
Avidin
pS/T/Y peptides
Avidin
Chemical cleavage
LC-MS phosphoproteome
LC-MS kinome
