# Supplementary figures and images for: Integrated proteome and phosphoproteome analysis of gastric adenocarcinoma reveals molecular signatures capable of stratifying patient outcome
Source: Mol Oncol. 2022 Dec 29;17(2):261–83. doi: 10.1002/1878-0261.13361 (PMC9892830; doi:10.1002/1878-0261.13361)

A

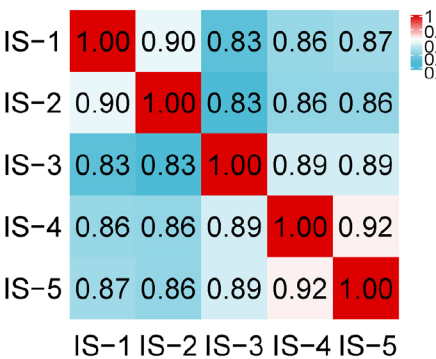

B

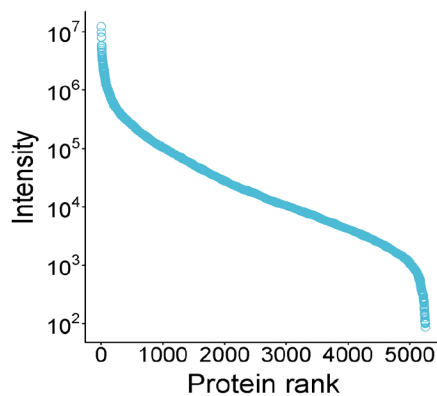

C

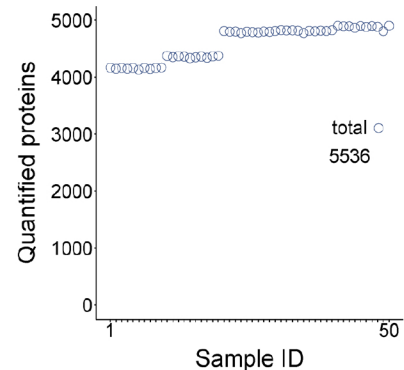

D

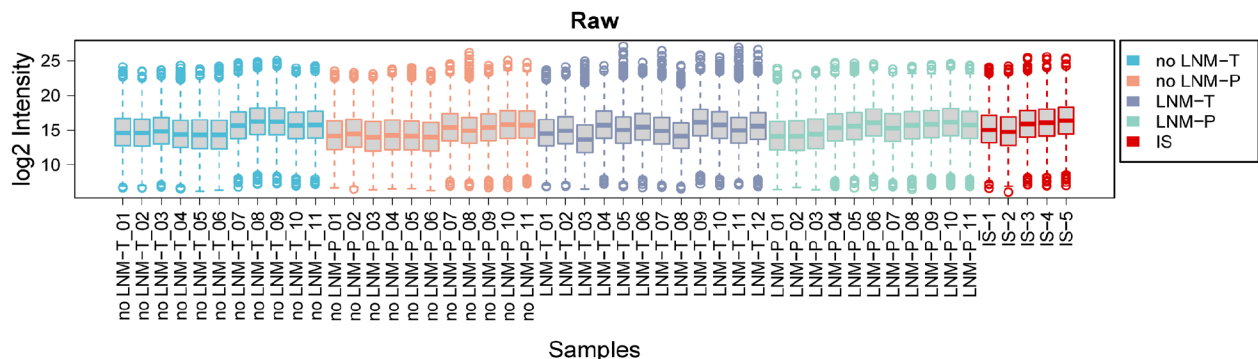

E

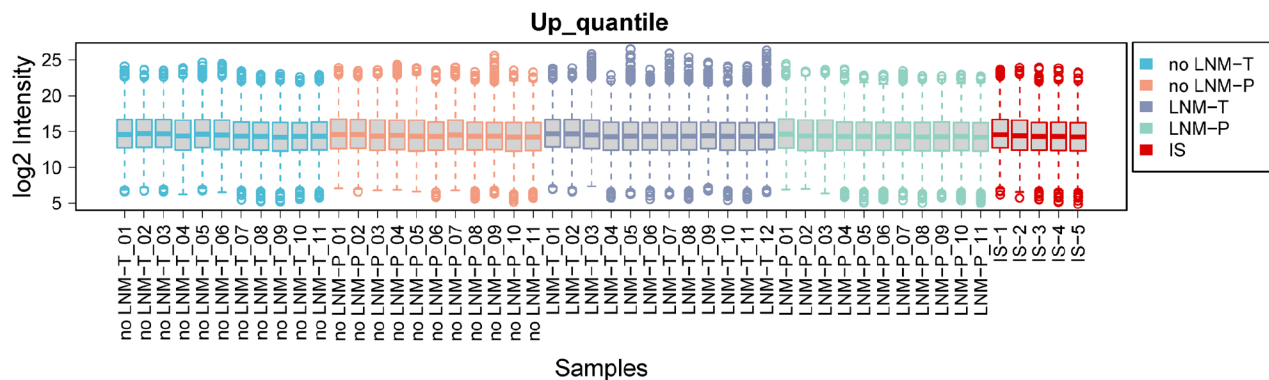

F

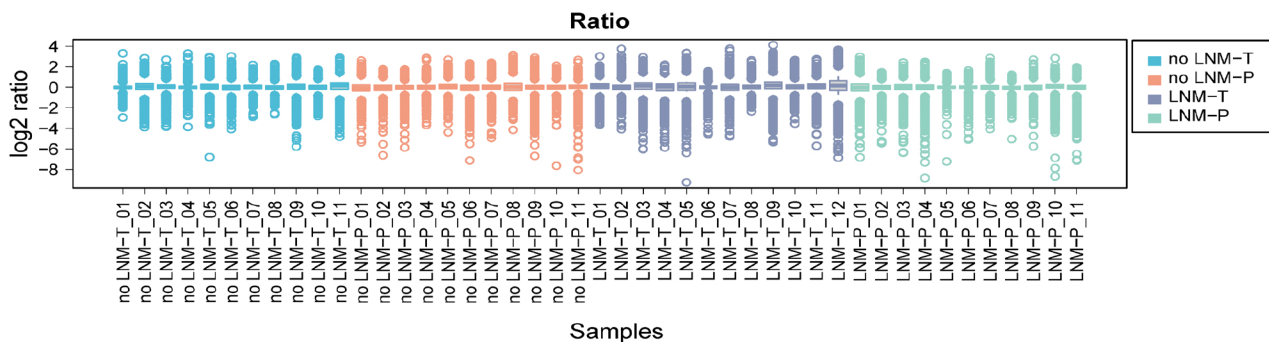

Supplement: Supplementary file 2 — Fig. S2. Proteomic data assessment and preprocessing. [file MOL2-17-261-s006.pdf]

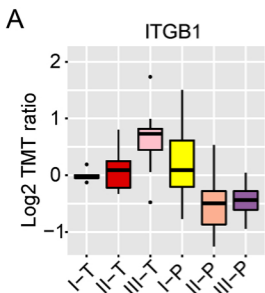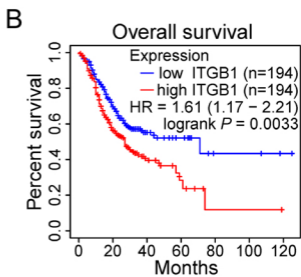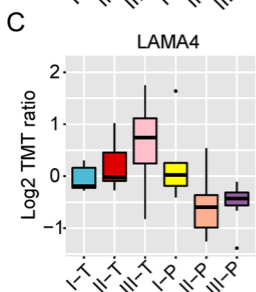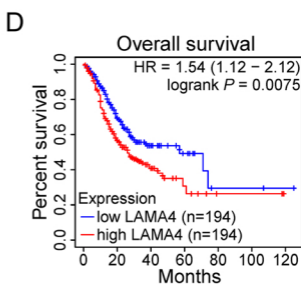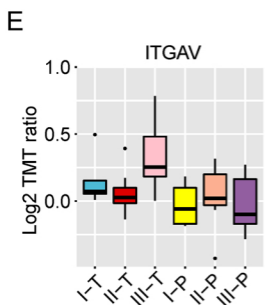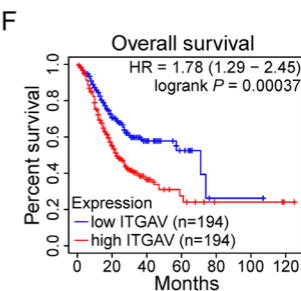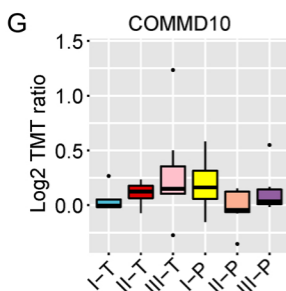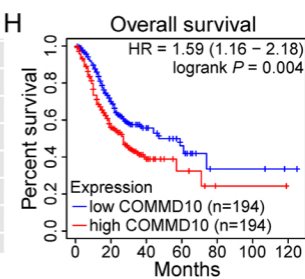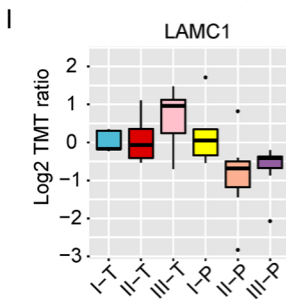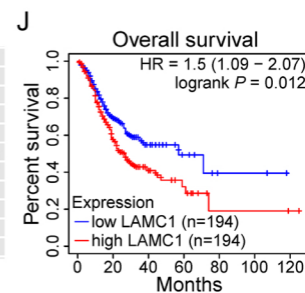

Supplement: Supplementary file 3 — Fig. S3. Overall survival analysis of hub proteins. [file MOL2-17-261-s010.pdf]

# Tumor LNM vs no LNM

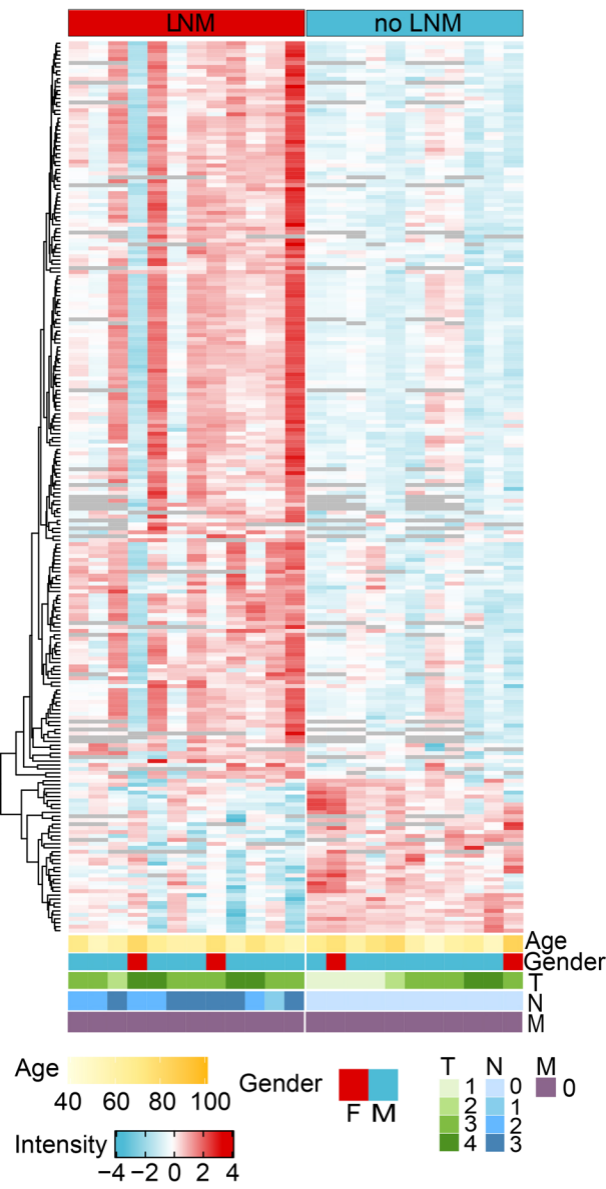

Supplement: Supplementary file 4 — Fig. S4. Heatmap of significantly DEPs in primary tumor from GAC patients in Plasma Proteome Database (PPD). [file MOL2-17-261-s005.pdf]

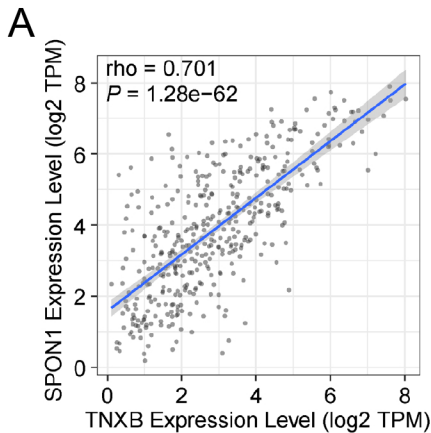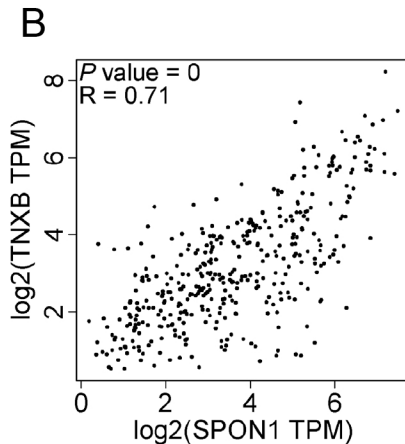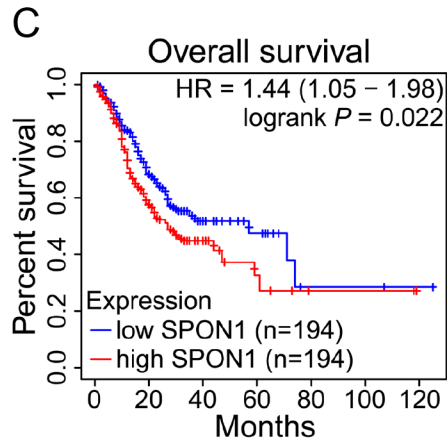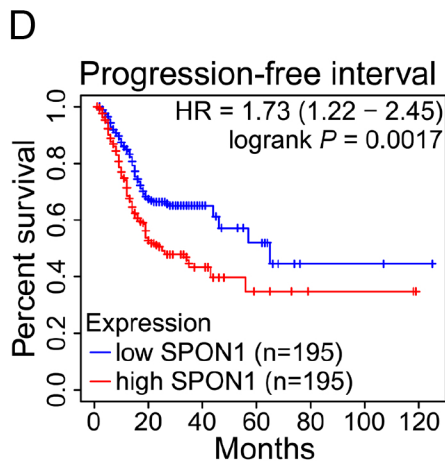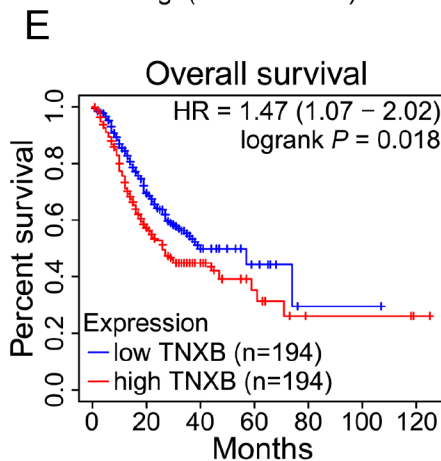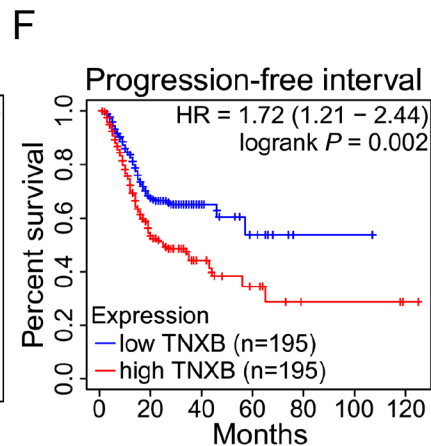

Supplement: Supplementary file 5 — Fig. S5. (A) Correlation analysis of SPON1 and TNXB expression at transcriptional level in TIMER 2.0 dataset. (B) Correlation expression analysis of SPON1 and TNXB in GEPIA dataset. (C–D) Overall survival (C) and progression‐free interval (D) of GAC patients using Kaplan–Meier estimator stratified by SPON1 expression, using 50% as the expression cutoff. (E–F) Overall survival (E) and progression‐free interval (F) of GAC patients using Kaplan–Meier estimator stratified by TNXB expression, using 50% as the expression cutoff. [file MOL2-17-261-s008.pdf]

A

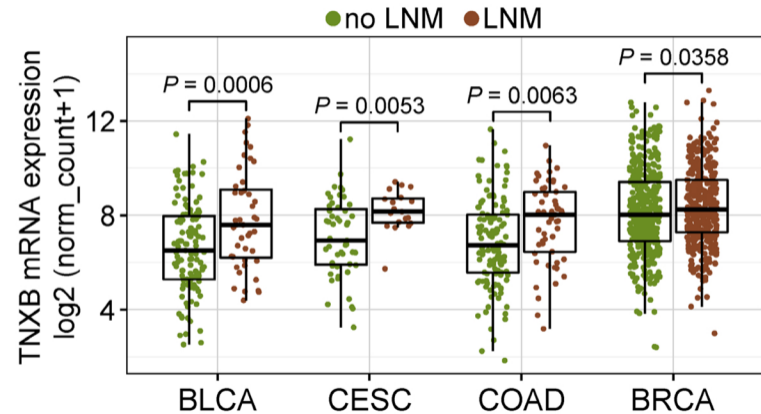

B

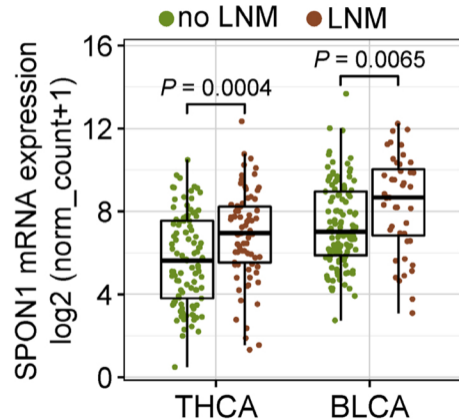

Supplement: Supplementary file 6 — Fig. S6. (A–B) mRNA expression of TNXB (A) and SPON1 (B) in other types of cancer from TCGA database between patients with only primary tumor versus patients with LNM. [file MOL2-17-261-s009.pdf]

A

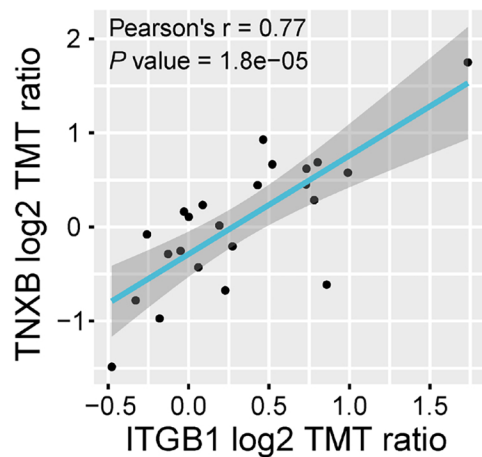

B

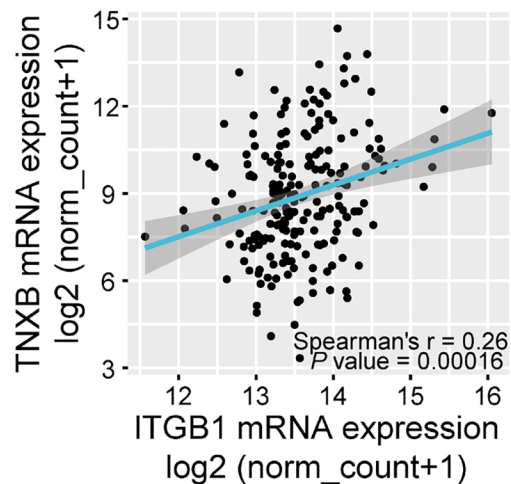

C

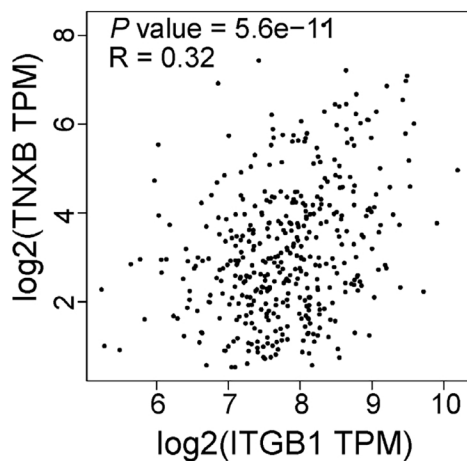

D

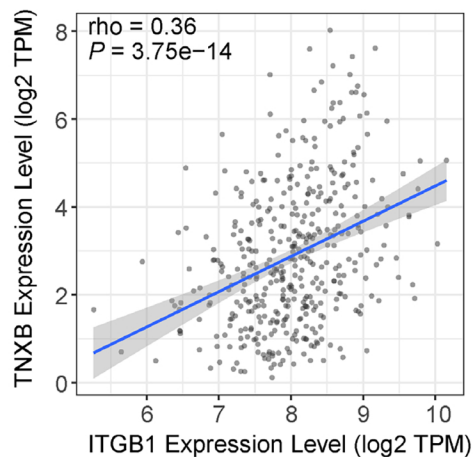

Supplement: Supplementary file 7 — Fig. S7. (A–D) Correlation expression analysis of ITGB1 and TNXB at protein level (A), transcription level in TCGA dataset (B), GEPIA (C), and TIMER 2.0 (D). [file MOL2-17-261-s003.pdf]
