# Supplementary material for: Integrated proteome and phosphoproteome analysis of gastric adenocarcinoma reveals molecular signatures capable of stratifying patient outcome
Source: Mol Oncol. 2022 Dec 29;17(2):261–83. doi: 10.1002/1878-0261.13361 (PMC9892830; doi:10.1002/1878-0261.13361)
Supplement: Supplementary file 8 — Fig. S8. (A–B) Correlation of TNXB, SPON1 expression, and infiltrating immune cells in gastric adenocarcinoma based on ITMER 2.0 dataset, including macrophages, neutrophils, myeloid dendritic cells (A), and T cells CD4+, CD8+, B cells (B). (C) Correlation of TNXB and SPON1 with immune cell‐specific marker genes at the protein level. * P < 0.05, ** P < 0.01, *** P < 0.001, n.s. P > 0.05. [file MOL2-17-261-s012.pdf]

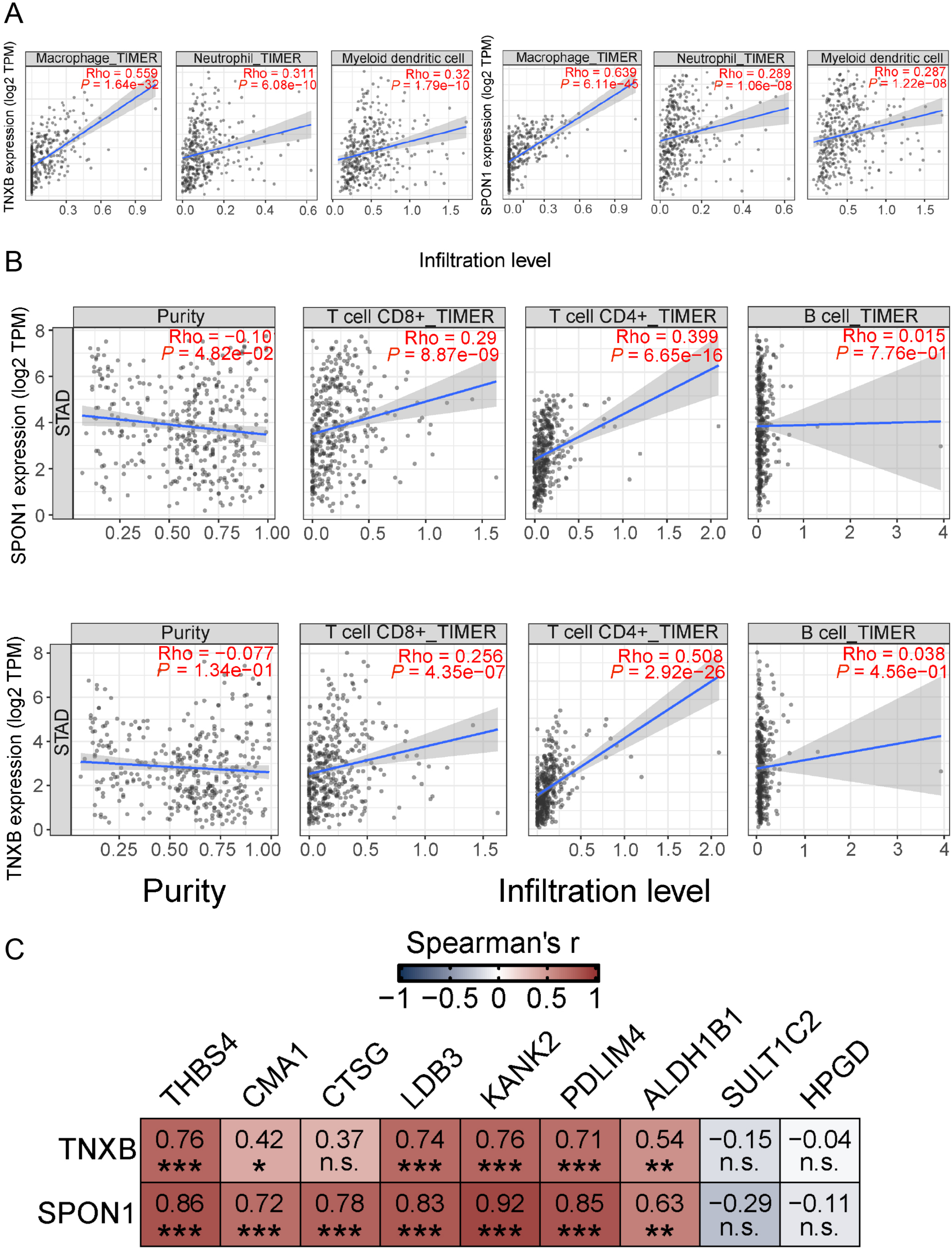
A
TNXB expression (log2 TPM)
Macrophage_TIMER
Rho = 0.559
P = 1.64e−32
Neutrophil_TIMER
Rho = 0.311
P = 6.08e−10
Myeloid dendritic cell
Rho = 0.32
P = 1.79e−10
SPON1 expression (log2 TPM)
Macrophage_TIMER
Rho = 0.639
P = 6.11e−45
Neutrophil_TIMER
Rho = 0.289
P = 1.06e−08
Myeloid dendritic cell
Rho = 0.287
P = 1.22e−08
Infiltration level
B
SPON1 expression (log2 TPM)
STAD
Purity
Rho = −0.10
P = 4.82e−02
T cell CD8+_TIMER
Rho = 0.29
P = 8.87e−09
T cell CD4+_TIMER
Rho = 0.399
P = 6.65e−16
B cell_TIMER
Rho = 0.015
P = 7.76e−01
TNXB expression (log2 TPM)
STAD
Purity
Rho = −0.077
P = 1.34e−01
T cell CD8+_TIMER
Rho = 0.256
P = 4.35e−07
T cell CD4+_TIMER
Rho = 0.508
P = 2.92e−26
B cell_TIMER
Rho = 0.038
P = 4.56e−01
Purity
Infiltration level
C
Spearman's r
−1 −0.5 0 0.5 1
THBS4
CMA1
CTSG
LDB3
KANK2
PDLIM4
ALDH1B1
SULT1C2
HPGD
TNXB
0.76 *** 0.42 * 0.37 n.s. 0.74 *** 0.76 *** 0.71 *** 0.54 ** −0.15 n.s. −0.04 n.s.
SPON1
0.86 *** 0.72 *** 0.78 *** 0.83 *** 0.92 *** 0.85 *** 0.63 ** −0.29 n.s. −0.11 n.s.
